# Female-specific reliance on endothelial FoxO1 governs vascular resilience and metabolic adaptation to obesity

**DOI:** 10.1007/s10456-026-10093-1

**Published:** 2026-10-22

**Authors:** Alexandra Pislaru, Emilie Roudier, Omid Rezvan, Connor Breadmore, Minoosh Fathi, George Nader, Ibrahim AlZaim, Emmanuel Nwadozi, Gisang Lee, Ehsan Yavari, Aly Fawzy, Eric Rullman, Joanna Kalucka, Thomas Gustafsson, Martina Rudnicki, Tara L. Haas

**Affiliations:** 1 Department of Biology, York University, Toronto, M3J 1P3 Canada; 2 School of Kinesiology and Health Science & Muscle Health Research Centre, York University, Toronto, M3J 1P3 Canada; 3 Department of Biomedicine, Aarhus University, Høegh-Guldbergsgade 10, 8000 Aarhus C, Denmark; 4 Steno Diabetes Center Aarhus, Aarhus University Hospital, Aarhus, Denmark; 5 Department Laboratory Medicine, Clinical Physiology, Karolinska Institutet and Department of Clinical Physiology, Karolinska University Hospital, Stockholm, Sweden; 6 UCL Institute of Ophthalmology, 11-43 Bath Street London EC1V 9EL, London, UK

**Keywords:** Angiogenesis, Endothelial, FoxO1, Adipose tissue, Mitochondria, Transcriptome profiling

## Abstract

Sex differences in adipose tissue vascular remodeling critically influence susceptibility to obesity-associated metabolic disease, yet the underlying mechanisms remain unclear. Here we identify Forkhead box O1 (FoxO1) as a sex-dependent regulator of endothelial function, with female endothelial cells (EC) expressing higher levels of FoxO1 and depending on it to maintain vascular remodeling and metabolic homeostasis. Accordingly, FoxO1 loss in females disrupted angiogenesis, and promoted adipocyte hypertrophy, weight gain and glucose dysregulation, whereas opposite effects have been reported in males. This phenotype was associated with a shift in the endothelial transcriptome toward a male-like state, consistent with loss of female protection against diet-induced metabolic dysfunction. Mechanistically, FoxO1 inhibition exerted time-dependent effects, with short-term suppression promoting proliferation and sustained inhibition reducing proliferation and inducing metabolic dysfunction. Although also observed in males, these effects were more pronounced in female EC, which also selectively exhibited mitochondrial dysfunction, increased oxidative stress and DNA damage accumulation, culminating in a senescence-like arrest. Antioxidant treatment partially rescued this phenotype, implicating oxidative stress as a driver of female-specific vulnerability. These findings reveal a sex-dependent divergence in FoxO1 function and identify endothelial FoxO1 as a determinant of opposing vascular and metabolic responses to obesogenic stress.

**Supplementary Information:**

The online version contains supplementary material available at https://doi.org/10.1007/s10456-026-10093-1.

## Introduction

White adipose tissue (WAT) is a highly plastic organ that expands in response to nutritional excess [1]. However, expansion of visceral adipose tissue is frequently accompanied by maladaptive remodeling, which represents a major risk factor for cardiometabolic disorders and increased all-cause mortality [2]. Biological sex is a variable that influences visceral WAT depot formation and function with females typically retaining healthier adipose tissue and experiencing less metabolic disruption compared to males, at equivalent levels of obesity [3, 4]. Although the biological underpinnings of these advantages are pleiotropic, a critical role for angiogenesis in facilitating physiological, rather than maladaptive, adipose tissue expansion is recognized [5, 6]. In line with this concept, visceral WAT angiogenesis is suppressed in male mice when challenged with high fat diet (HFD) [7–11], whereas female mice retain a superior angiogenic capacity [12]. Despite these observations, the molecular mechanisms that govern sex-specific differences in angiogenic capacity remain largely unresolved.

Endothelial cells (EC), which form the adipose microvasculature, are central to this process. Metabolic stress-induced endothelial dysfunction is a major contributor to impaired angiogenic responses in obesity [13–15]. Emerging studies have identified sex-specific differences in EC gene expression and behavior that shape cellular responses to a range of metabolic stressors including HFD [16–18]. Notably, adipose EC from HFD-fed female mice display a distinct transcriptomic profile characterized by an enhanced proliferative signature and increased stress resistance relative to male EC [18]. Defining the molecular pathways that sustain EC resilience, the ability of EC to preserve core physiological functions under metabolic stress, in females is therefore essential for understanding sex differences in vascular adaptation to metabolic stress.

Forkhead Box O1 (FoxO1) is a key regulator of EC biology. While it is indispensable for vascular development during embryogenesis [19, 20], in the adult endothelium FoxO1 enforces quiescence by suppressing proliferative pathways and glycolytic metabolism [21, 22]. Beyond this canonical role, FoxO1 has emerged as a key regulator of endothelial cell identity in human adipose tissue, where it displays high transcriptional activity, implicating a role in maintaining adipose endothelial homeostasis [23]. Consistent with its function as a gatekeeper of quiescence, long-term HFD provokes greater expression of endothelial cell FoxO1, and the endothelial-specific FoxO1 deletion in male mice enhances angiogenesis and protects against diet-induced metabolic dysfunction [9, 24]. However, these findings derive largely from male models or sex-unspecified systems, leaving it unclear whether FoxO1 exerts similar functions in female EC. This represents a critical gap, given that adipose EC from females exhibit greater proliferative capacity and stress resilience despite higher FoxO1 expression [18], suggesting a fundamentally distinct regulatory context. These observations raise the possibility that FoxO1 serves divergent, sex-dependent roles in endothelial adaptation to metabolic stress, underscoring the need to directly test its function in females.

Here, we tested the hypothesis that FoxO1 is required to maintain endothelial resilience in females. Using complementary genetic models and pharmacological inhibition approaches, we show that FoxO1 loss induces a female-specific vulnerability to metabolic stress, revealing a divergence in FoxO1 function between sexes and identifying FoxO1 as a key regulator of sex-dependent vascular and metabolic adaptation to obesity.

## Results

### FoxO1 levels and activity are higher in female compared to male EC

**Fig. 1:**
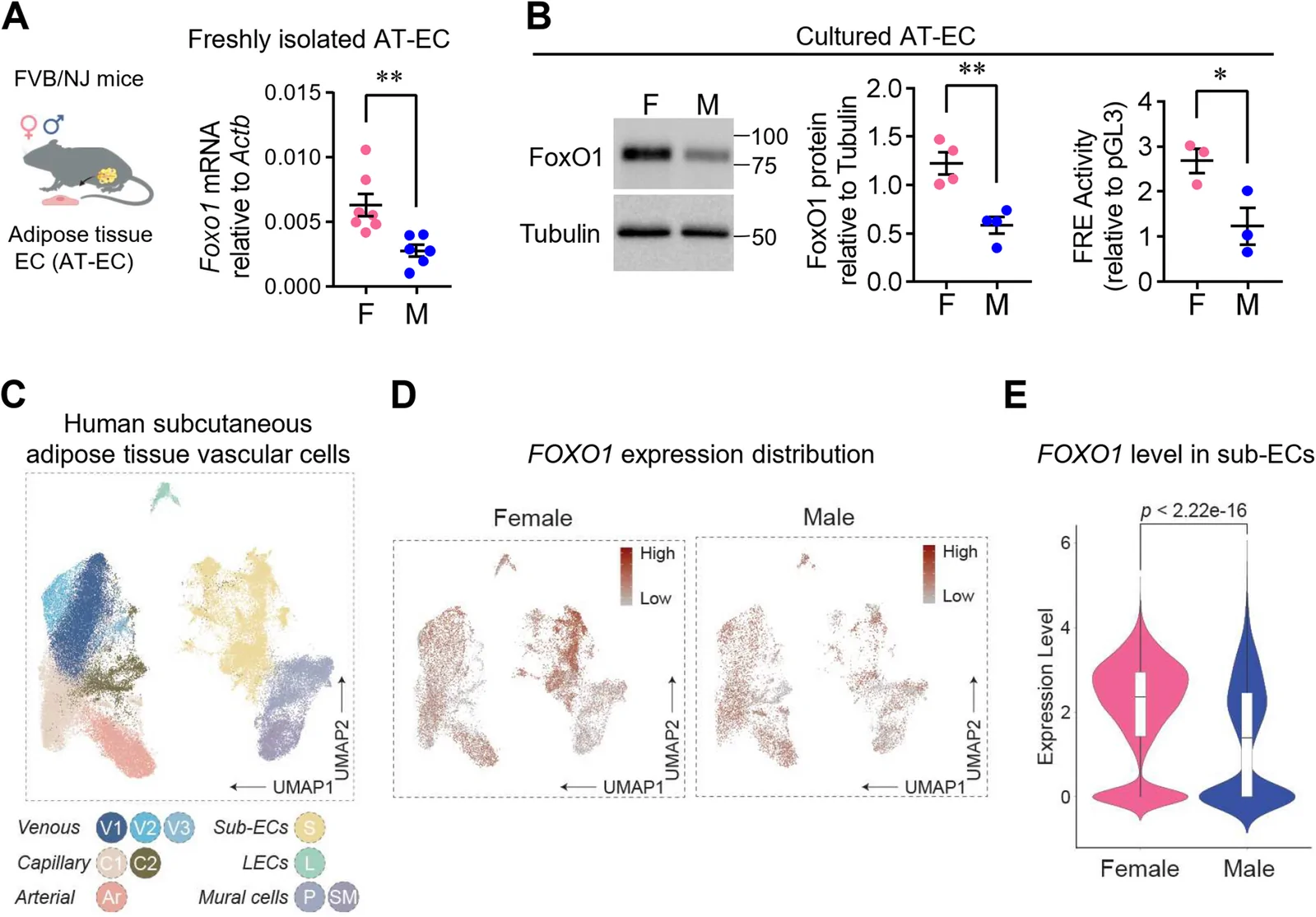
FoxO1 mRNA, protein and activity are intrinsically higher in female EC. **A*** Foxo1* mRNA levels relative to *Actb* in adipose EC freshly isolated from standard chow-fed FVB/NJ mice (Female n=7, Male n=6). **B** Cultured female (F) and male (M) mouse adipose EC were assessed for whole cell FoxO1 protein (normalized to tubulin) (n=4) and Forkhead-response-element (FRE)-luciferase activity (n=3). Data points reflect individual mice or independent plates of cultured cells and are plotted with the group mean ± SEM. Data were analyzed by Students t-test. *P<0.05; **P<0.01. **C-E** *FOXO1* expression was analysed in the human subcutaneous adipose tissue vascular cell atlas [23]. **C** UMAP of 68,503 vascular cells from the human subcutaneous adipose tissue vascular cell atlas, identifying ten distinct vascular cell populations. **D** Feature plots show *FOXO1* expression in vascular cells from non-obese female (left panel) and male (right panel) donors. Colour intensity reflects normalized *FOXO1* expression (dark red, high; grey, low). **E** Violin plot comparing *FOXO1* expression in subcutaneous endothelial cells (sub-ECs) from non-obese female and male donors. Violin plots show the distribution of single-cell expression values, with the embedded boxplot indicating the median (center line), interquartile range (box), and minimum and maximum values (whiskers). Statistical significance was assessed using a Wilcoxon rank-sum test. Abbreviations: ECs, endothelial cells; LECs, lymphatic endothelial cells; UMAP, Uniform Manifold Approximation and Projection Schematic created in BioRender. Rudnicki, M. (2026) https://BioRender.com/xmttcqs

We previously reported that *Foxo1* mRNA levels are elevated in adipose EC from HFD-fed female mice compared with males [18]. To investigate the scope of this sex difference in FoxO1 expression, we first assessed *Foxo1* mRNA in adipose EC isolated from wild-type mice maintained on standard chow (Fig. 1A). *Foxo1* transcript levels were approximately two-fold higher in freshly isolated female versus male adipose EC (Fig. 1A). This sex difference was also detected at the protein level in murine adipose EC maintained in cell culture for numerous passages (Fig. 1B), indicating that elevated FoxO1 expression in female EC is a stable, cell-intrinsic feature. Furthermore, transcriptional activity of a forkhead response element (FRE)-luciferase reporter construct was significantly higher in the female compared to male EC (Fig. 1B), providing functional evidence of enhanced FoxO1 activity and supporting a cell-autonomous role for FoxO1 in shaping sex-specific EC phenotypes. Notably, this sex difference in *FOXO1* expression was also evident in human adipose endothelial cells from non-obese individuals. Analysis of the human subcutaneous adipose tissue vascular cell atlas [23], showed that the Sub-EC cluster exhibited the greatest *FOXO1* expression among the vascular cell populations (Fig. 1C, D). Sex-stratified analysis of this cluster revealed significantly higher *FOXO1* mRNA in female than in male EC (Fig. 1E). Thus, our findings demonstrate a conserved and stable female-biased pattern of FoxO1 expression in adipose EC.

### EC-FoxO1 depletion disrupts angiogenesis and drives adipose dysfunction in HFD-fed female mice

**Fig. 2:**
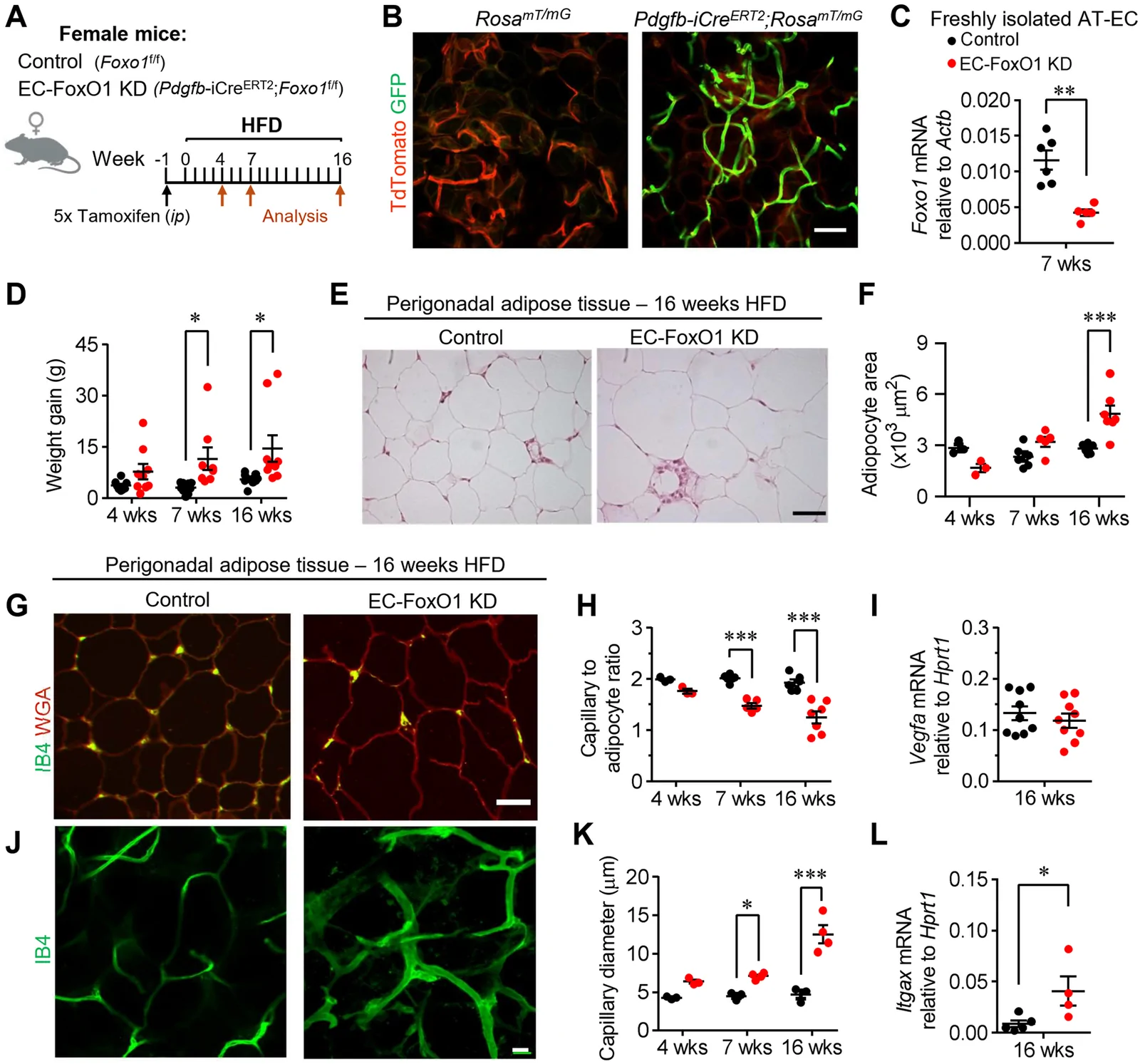
EC-FoxO1 knockdown represses adipose angiogenesis and induces capillary dysmorphia in female mice. **A** Female Control and EC FoxO1 KD mice were fed HFD for 4, 7 or 16 weeks. **B** pWAT images of tamoxifen-injected *Rosa*^(mT/mG)^ and *Pdgfb*-iCre^ER;T2^;*Rosa*^mT/mG^ mice illustrate the endothelial-specificity of Cre recombinase activity. Green: GFP; Red: tdTomato. Scale bar=50 µm. **C*** Foxo1* mRNA relative to *Actb* (2^−ΔCT^) in female EC isolated from white adipose of 7-week HF fed Control and EC-FoxO1 KD mice (n=6). **D** Total weight gain of female mice over the 16 weeks HFD. **E** Hematoxylin and eosin-stained images of pWAT from 16-week HFD-fed female mice. Scale bar=50 µm. **F** Adipocyte cross-sectional areas. **G** pWAT adipose sections stained with *G.simplicifolia* lectin-FITC (IB4; green) and Rhodamine Wheat germ agglutinin (WGA, red) to label capillaries and basement membranes, respectively. Scale bar=100 µm. **H** Quantification of capillary to adipocyte ratios. **I** *Vegfa* mRNA relative to *Hprt1* in female pWAT (16-week HFD). **J** *G. simplicifolia* lectin-FITC (green) imaging of capillaries in whole-mount pWAT. Scale bar=20 µm. **K** Capillary diameters in female mice after 4, 7 or 16-weeks HFD. **L*** Itgax* mRNA relative to *Hprt1* (2^−ΔCT^) from pWAT of 16-week HFD-fed female mice (n=5). Data points reflect individual mice, and are plotted with the group mean ± SEM. Data were analyzed by Students t-test or by 2-way ANOVA with Sidak post-hoc testing. *P<0.05; **P<0.01; ***P<0.001. Schematic created in BioRender. Rudnicki, M. (2026) https://BioRender.com/xmttcqs

To investigate whether the higher level of FoxO1 in female EC is associated with resistance to diet-induced obesity and its complications, we generated EC-specific knockdown (EC-FoxO1 KD) female mice using the tamoxifen-inducible, endothelial-selective *Pdgfb-iCreER*^*T2*^*:Foxo1*^*f/f*^ mouse model, as previously described [9], and exposed them to a HFD for 16 weeks (Fig. 2A). Endothelial specificity of the *Pdgfb-iCreER*^*T2*^ driver was confirmed using the *Rosa26-mT/mG* (mTmG) reporter line, which showed robust GFP expression exclusively in EC following tamoxifen administration in adult mice (Fig. 2B; Fig. S1A). Gene expression analysis of adipose EC isolated 8 weeks post-tamoxifen (and after 7 weeks of HFD) revealed a 58% reduction in *Foxo1* mRNA in EC-FoxO1 KD females compared to Cre-negative controls (Fig. 2C). This was consistent with the 60% reduction in *Foxo1* mRNA in adipose EC of EC-FoxO1 KD males compared to their respective controls (Fig. S1B).

Under HFD feeding, EC-FoxO1 KD females exhibited accelerated weight gain, which emerged as early as 7 weeks after diet initiation (Fig. 2D; Table S1). WAT mass also significantly increased (Table S1) and histological analysis of pWAT revealed adipocyte hypertrophy after 16 weeks of HFD, with adipocyte size increasing approximately 1.7-fold relative to controls (Fig. 2E, F). In parallel, EC-FoxO1 KD females exhibited a reduction in capillary content within pWAT (*P:*0.0099 for overall effect of *Foxo1* deletion), with ~1.3-fold and ~1.5-fold reductions in capillary density observed after 7 and 16 weeks of HFD, respectively (Fig. 2G, H). These findings are in striking contrast to prior observations in EC-FoxO1 KD male mice fed HFD, which exhibited reduced weight gain, smaller adipocyte size and increased capillary number [9] (Fig. S1C-E). Notably, the angiogenic defect in females occurred despite comparable tissue *Vegfa* transcript levels between EC-FoxO1 KD and controls (Fig. 2I), suggesting a VEGFA-independent mechanism.

In addition to the reduced capillary density, *Foxo1* depletion in female EC was accompanied by capillary enlargement (*P:*0.0003 for overall effect of *Foxo1* deletion) (Fig. 2J,K), indicative of aberrant vascular remodeling. In EC-FoxO1 KD females, capillary diameters trended to increase after only 4-weeks HFD and showed significant expansion after 7 and 16 weeks (~ 1.7 and 2.7-fold above controls, respectively) (Fig. 2J,K; Fig. S2A). Although moderate capillary enlargement following EC-FoxO1 depletion has been reported in male mice [9] and was replicated here (Fig. S1F), this remodeling was exacerbated by sex. Average capillary diameters in EC-FoxO1 KD females reached 12.5 ± 1.2 µm compared to 8.8 ± 0.5 µm in males after 16 weeks of HFD (Fig. 2K; Fig. S1F). Moreover, vessels in EC-FoxO1 KD females frequently displayed aberrant morphologies, such as monolayer-like structures or glomerular-like formations (Fig. S2A), consistent with disorganized angiogenesis. We also observed elevated expression of the immune cell marker *Itgax* in pWAT of EC-FoxO1 KD females after 16 weeks of HFD (Fig. 2L), which may reflect increased adipose tissue inflammation [25]. Together, these findings support a model in which elevated FoxO1 levels in females are required to sustain proper angiogenic remodeling and promote healthy adipose expansion under obesogenic conditions.

### EC-FoxO1 depletion in females reduces capillarization across other metabolic tissues and disrupts systemic glucose metabolism

**Fig. 3:**
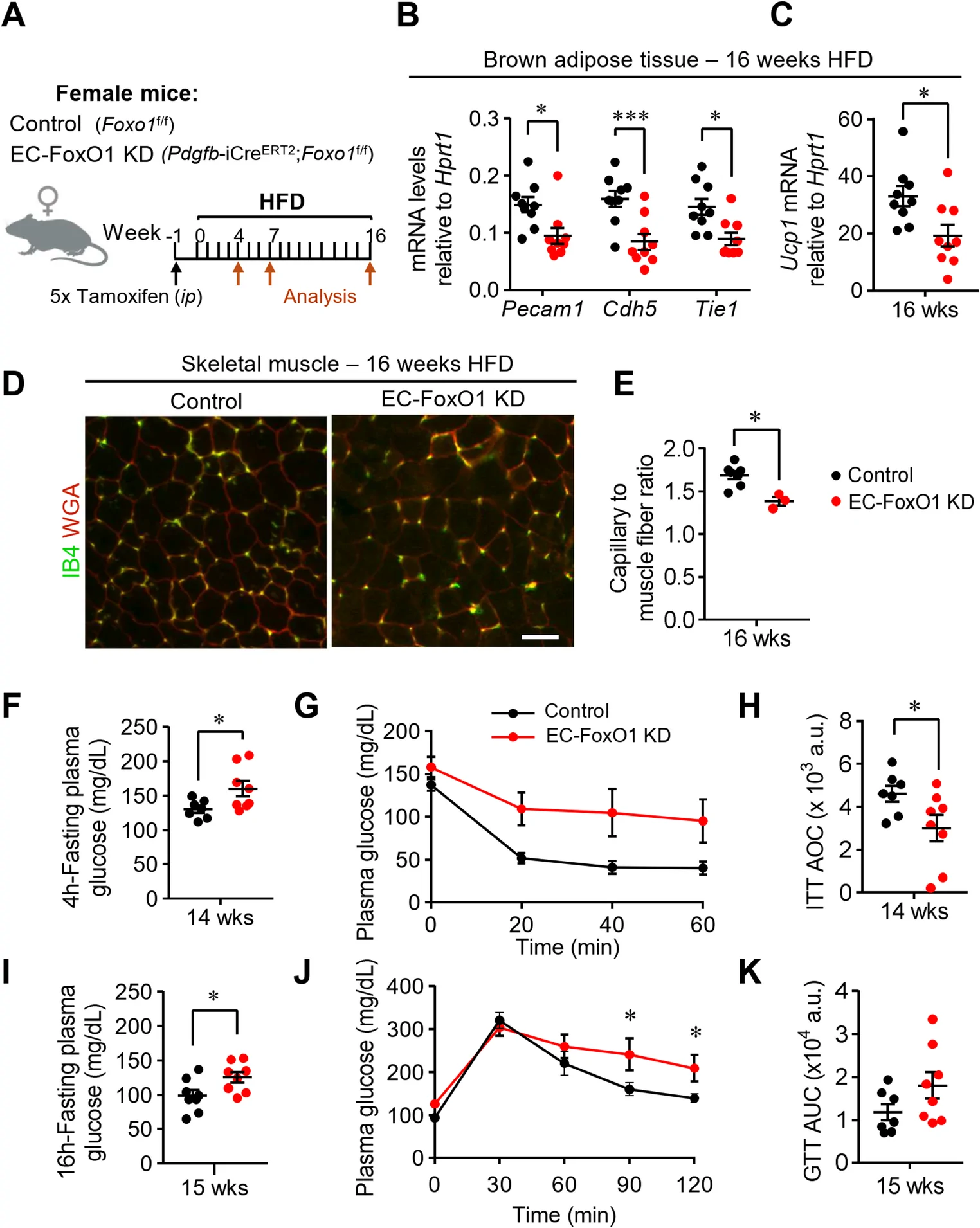
EC-specific knockdown of FoxO1 exerts broad vascular and metabolic disturbances in HFD-fed female mice. **A** Control and EC-FoxO1 KD female mice were fed HFD for 16 weeks. **B, C** Endothelial markers and *Ucp1* mRNA relative to *Hprt1* (2^−ΔCT^) from female brown adipose tissue (16-week HFD)(n=9). **D** Cross-sections of female EDL muscle stained with *G. simplicifolia* lectin-FITC (IB4, green) and Rhodamine Wheat Germ Agglutinin (WGA, red) to visualize capillaries and muscle fibers, respectively. Scale bar=100 µm. **E** Quantification of muscle capillary to muscle fiber ratios. **F–H** Insulin tolerance tests (ITT). **F** Plasma glucose levels of Control and EC-FoxO1 KD mice after 4 h fasting. **G** Glucose levels during ITT in Control and EC-FoxO1 KD mice. **H** ITT area over the curve. **I-K** Glucose tolerance tests (GTT). **I** Plasma glucose levels of HF-fed Control and EC-FoxO1 KD mice after 16-h fasting. **J** Glucose levels during GTT in Control and EC-FoxO1 KD mice. **K** GTT area under the curve. Data points reflect individual mice and are plotted with the group mean ± SEM. Data were analyzed by Students t-test. *P<0.05; ***P<0.001. Schematic created in BioRender. Rudnicki, M. (2026) https://BioRender.com/gtbkoy3

We next examined whether the vascular defects observed in WAT extended to other metabolically relevant organs of EC-FoxO1 KD in females following HFD feeding (Fig. 3A). The brown adipose tissue (BAT) of EC-FoxO1 KD females displayed significantly reduced expression of endothelial markers *Pecam1*, *Cdh5* and *Tie1* compared to controls after 16 weeks HFD, indicative of decreased vascular content (Fig. 3B). This effect was detected as early as after 7 weeks of HFD, as assessed by whole-mount capillary imaging (Fig. S2B, C). In addition, EC-FoxO1 KD females fed a HFD for 16 weeks exhibited significantly lower expression of *Ucp1*, a hallmark thermogenic gene of functional BAT, when compared to controls (Fig. 3C), indicating a shift toward a ‘‘whitening’’ phenotype consistent with dysfunctional BAT remodeling [8]. Histological analysis of skeletal muscle revealed an ~18% reduction in the capillary-to-muscle fiber ratio in HFD-fed EC-FoxO1 KD females compared to controls (Fig. 3D,E), indicating that FoxO1 is required to maintain capillary networks across multiple metabolic tissues in female mice under obesogenic conditions. As muscle capillarity is an important predictor of insulin-mediated glucose disposal in mice and humans [26, 27], we next analyzed whole-body glucose regulation in HFD-fed EC-FoxO1 KD and control female mice. EC-FoxO1 KD females had elevated fasting glucose levels (Fig. 3F, I) and reduced insulin sensitivity, as determined by insulin tolerance testing (Fig. 3G, H), while glucose tolerance was moderately impaired (Fig. 3J, K). It is noteworthy that these vascular and metabolic impairments contrast with the previously reported increases in BAT and skeletal muscle capillarization and improved glucose regulation in HFD-fed male EC-FoxO1 KD mice [9], thereby reinforcing a sexual dimorphic, pan-tissue role for EC FoxO1 in metabolic regulation.

### EC-FoxO1 deletion also disrupts vascular content and metabolic homeostasis in females under low-fat diet conditions

The elevated levels of FoxO1 observed in female EC from chow-fed mice and in cultured EC suggest that FoxO1 performs constitutive, physiologically protective functions in female EC. We therefore reasoned that deletion of EC-FoxO1 should elicit detectable vascular and metabolic defects even in the absence of obesogenic stress. Accordingly, we examined vascular and metabolic characteristics of EC-FoxO1 KD females maintained on a low-fat diet (LFD) for 16 weeks (Fig. S3A). EC-FoxO1 KD females displayed a modest but significant increase in weight gain compared to controls, which became evident only after 16 weeks (Fig. S3B). Similarly, pWAT from LFD-fed EC-FoxO1 KD mice recapitulated several pathological features observed under HFD-fed conditions, including adipocyte hypertrophy (Fig. S3C, D), elevated expression of the immune marker *Itgax* (Fig. S3E) and reduced capillary number (~ 1.6-fold decrease, Fig. S3F, G), in the absence of changes in tissue *Vegfa* expression (Fig. S3H). Capillaries from LFD-fed EC-FoxO1 KD females also exhibited diameter enlargement (2.8-fold increase, Fig. S3I, J) relative to controls. Approximately ~12% of pWAT capillaries in LFD-fed EC-FoxO1 KD females exceeded 20 µm in diameter, compared to fewer than 4% reaching that size in males (Fig. S3K, L), indicating a pronounced, sex-specific, and diet-independent vascular phenotype.

Similar to the outcomes observed in HFD-fed mice, EC-FoxO1 depletion in LFD-fed mice also reduced endothelial markers and *Ucp1* expression in the BAT (Fig. S4A, B), concomitant with diminished capillary number in the skeletal muscle (Fig. S4C, D). At the systemic level, LFD-fed EC-FoxO1 KD females exhibited impaired insulin sensitivity (Fig. S4E, F), while glucose tolerance was unaffected (Fig. S4G, H). Elevated plasma glucose levels were detected only under short-term fasting conditions (Fig. S4I, J), rather than the persistent hyperglycemia seen under HFD conditions (Fig. 3F, I).

Altogether, these findings reveal a sex-specific requirement for EC-FoxO1 in maintaining vascular and metabolic homeostasis in females, as EC-FoxO1 deletion exerts effects on angiogenesis, adipose morphology and systemic metabolic health that are opposite to those observed in males. These effects are evident in LFD-fed mice but are further accentuated by long-term HFD exposure.

### FoxO1 KD induces a sex-biased and dysfunctional shift in the EC transcriptome

**Fig. 4:**
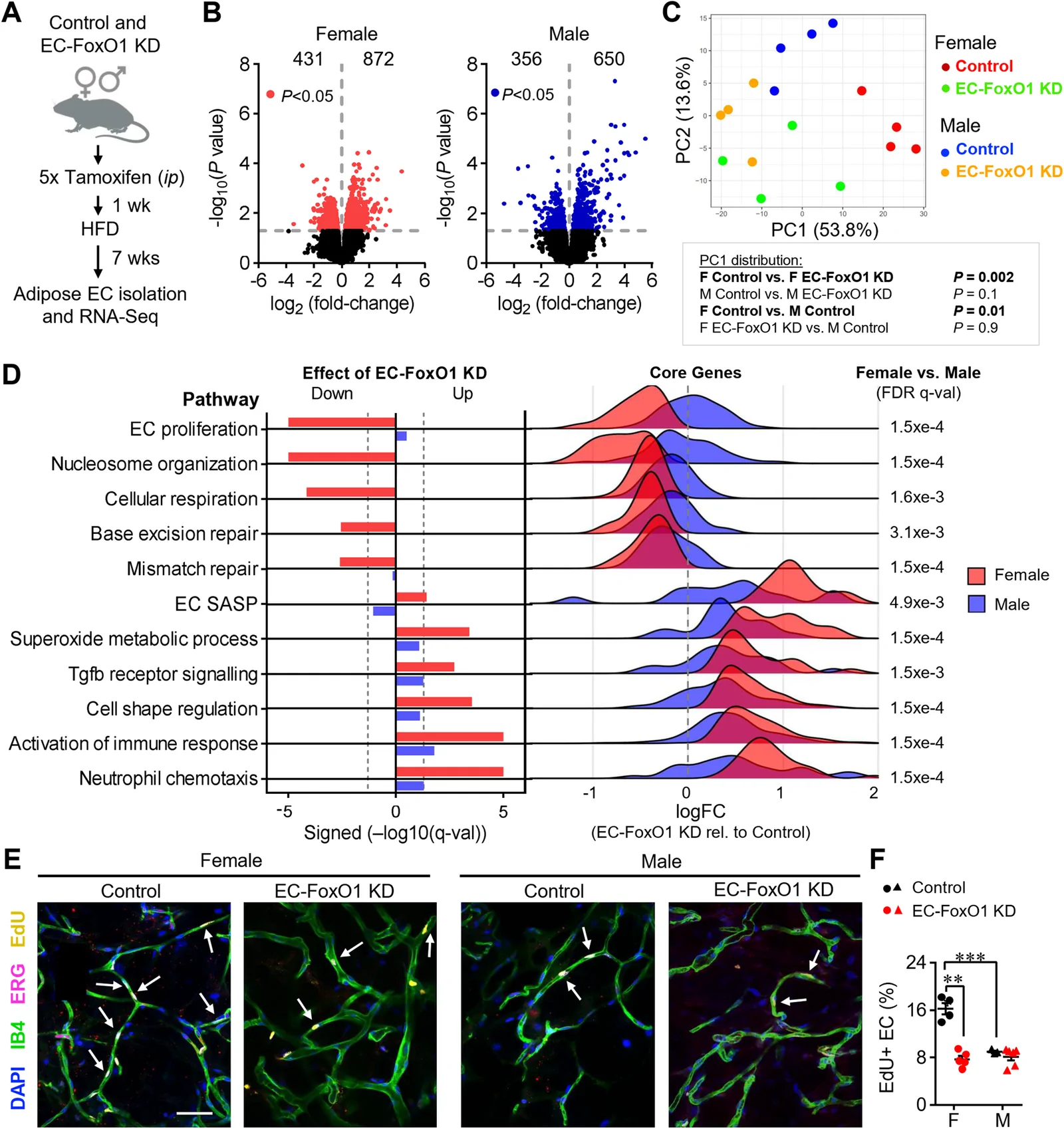
Endothelial FoxO1 knockdown in HFD mice elicits sex-distinct transcriptomic reprogramming and impairs proliferative capacity only in female EC. **A** Female and male Control and EC-FoxO1 KD mice were HFD-fed for 7-weeks then RNA was extracted from freshly isolated adipose EC and analyzed by RNA-seq (n=4 per group). **B** Volcano plots illustrating the expression profile of genes in EC-FoxO1 KD vs. Control female and male adipose EC. A total of 1,323 and 1,006 DEGs were identified in female and male EC, respectively, *P* < 0.05 (indicated by the dashed horizontal line). **C** Principal component analysis (PCA) of EC gene expression from each mouse, based on female DEG. 2-way ANOVA established main effects of Sex and Genotype on PC1 values. Results of Sidak post-hoc testing of group differences in PC1 are displayed below the PCA graph. For each sex, GSEA was performed (EC-FoxO1 KD vs. Control) to identify pathways altered by FoxO1 KD. **D** Selected pathways significant in female EC are highlighted, with corresponding values for these pathways in male EC. Sign reflects direction of pathway enrichment (up or down), and bar magnitude reflects statistical significance (-log10(FDR q-values)). Dashed lines mark the FDR q-value threshold of 0.05. The logFC values for core enrichment genes within each of these pathways are displayed in the corresponding ridge plot. Sex differences in the fold-change values of these core genes from each pathway was assessed (Wilcoxon, reporting the FDR adjusted q-value). EC proliferation and EC SASP pathways were custom as described previously [15], while all others represent Gene Ontology Biological Processes and WikiPathways. **E** Whole-mount confocal imaging of visceral adipose Control and EC-FoxO1 KD mice following 7-wk HFD. EdU incorporation (Gold) in EC was detected by co-staining with *G. simplificolia* (IB4, green) to detect capillaries, ERG (red) to detect EC nuclei and DAPI (blue) to label all nuclei. Scale bar=50 µm. **F** EdU-positive EC were quantified as a percentage of total EC nuclei. Data points reflect individual mice and are plotted with the group mean ± SEM (Female Control n=4, Female EC-FoxO1 KD n=5, Male Control n=3, Male EC-FoxO1 KD n=6). Circles denote females, triangles denote males. Data were analyzed by 2-way ANOVA with Sidak post-hoc testing. **P<0.01; ***P<0.001. Schematic created in BioRender. Rudnicki, M. (2026) https://BioRender.com/gtbkoy3

To elucidate the molecular programs underlying the sex-distinct effects of EC-FoxO1 KD, we conducted transcriptomic profiling of adipose EC isolated from male and female EC-FoxO1 KD and control mice. To enhance phenotypic divergence between sexes, mice were conditioned with HFD for 7 weeks prior to EC isolation (Fig. 4A). EC-FoxO1 KD resulted in 1,303 differentially expressed genes (DEG) in female EC and 1,006 DEGs in male EC (*P*_Adj_ <0.05) (Fig. 4B). Principal component analysis (PCA) revealed a significant separation between female control and EC-FoxO1 KD samples along PC1, whereas male control and EC-FoxO1 KD samples were not significantly distinct. Female and male control EC were significantly distinct along PC1. However, EC-FoxO1 KD shifted female EC toward the control male profile, such that they no longer differed from males (Fig. 4C). Gene set enrichment analysis (GSEA) further demonstrated that EC-FoxO1 depletion perturbed a broader range of biological pathways in females than in males (Table S2), highlighting a heightened transcriptomic dependency on FoxO1 in female EC. Specifically, EC-FoxO1 KD led to significant suppression of gene programs associated with proliferation, cellular respiration, nucleosome organization, and DNA repair only in female EC (Fig. 4D; Table S2). Furthermore, EC-FoxO1 KD in female mice selectively induced gene programs related to superoxide metabolism, endothelial senescence associated secretory phenotype (SASP) and superoxide metabolism (Fig. 4D; Table S2). EC-FoxO1 KD enriched pathways related to TGFβ receptor signaling, cell shape regulation, activation of immune responses and inflammation (ie. neutrophil chemotaxis) in both sexes, but in each case with a greater magnitude in females (Fig. 4D; Table S2). This exaggerated influence of EC-FoxO1 KD in females was further exemplified when comparing the effect of EC-FoxO1 KD on expression changes of the core genes represented within each ontology, all of which shifted to a greater extent in female than in male EC (Fig. 4D). Thus, these transcriptomic profiles identify FoxO1 as a key regulator of female endothelial cellular homeostasis, as its loss exerts broader and more deleterious effects in female compared to male adipose EC.

Given the suppression of proliferative gene signatures following FoxO1 depletion in female EC, which is contrary to the established role of FoxO1 as an inhibitor of proliferation [21, 22, 28], we investigated whether this transcriptional shift translated into functional changes in endothelial proliferation in vivo. Assessment of EdU incorporation in adipose EC from HFD-fed male and female mice revealed a significant reduction in proliferating EC exclusively in EC-FoxO1 KD females compared with controls (Fig. 4E, F). These findings expose a female-specific requirement for FoxO1 in sustaining endothelial replication, challenging the prevailing view of FoxO1 solely as an inhibitor of cell proliferation.

### FoxO1 inhibition induces a time-dependent, sex-specific switch from pro- to anti-proliferative responses in cultured adipose EC

**Fig. 5:**
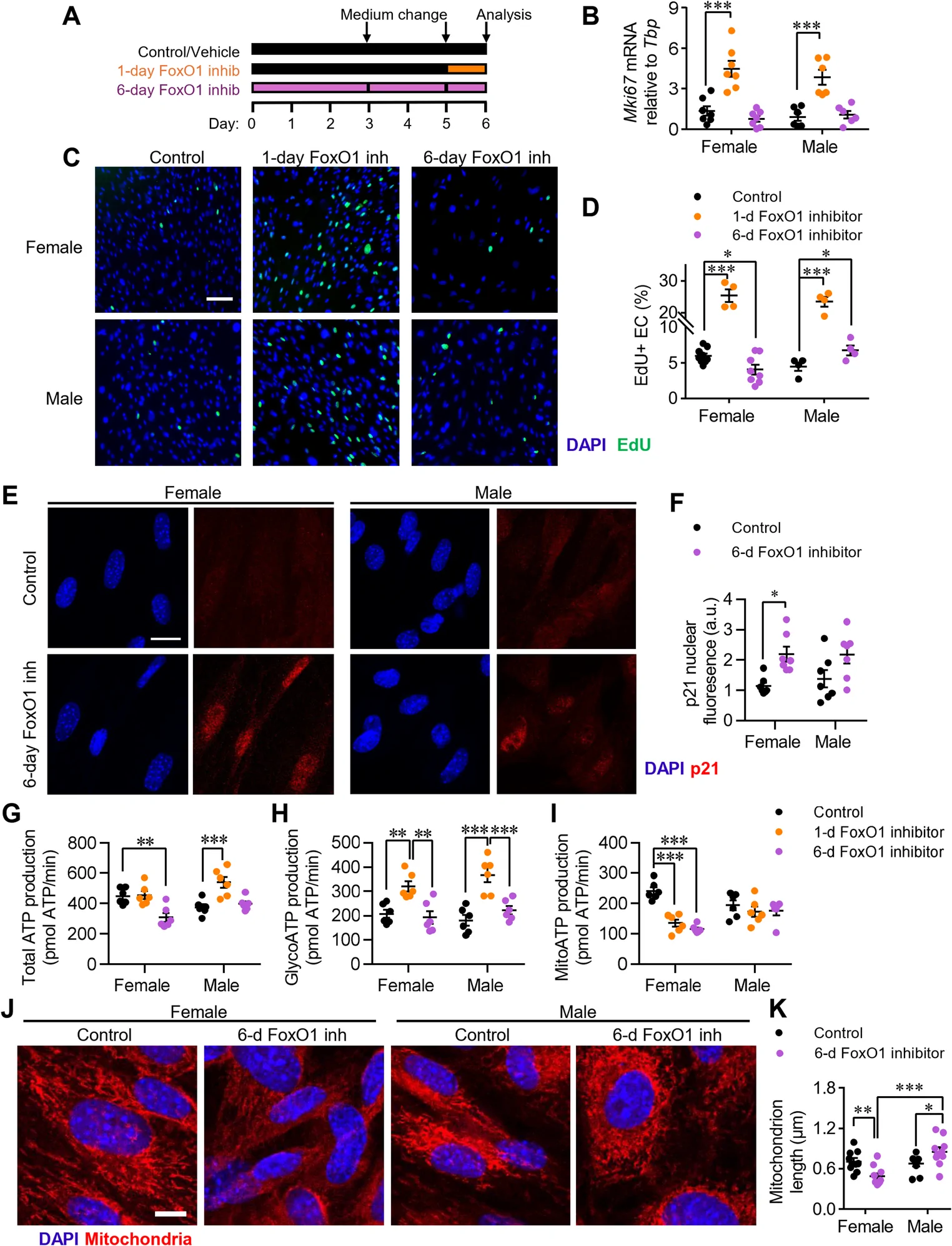
Sustained FoxO1 inhibition decreases EC proliferation but selectively impairs mitochondria in female EC in vitro. **A** Schematic of 1-day and 6-day FoxO1 inhibition (AS1842856, 1 µM) in cultured EC. **B** mRNA of *Mki67* following FoxO1 inhibitor treatment normalized to housekeeping *Tbp* (2^−ΔCT^) (Female n=7, Male n=6). **C** EdU incorporation in cultured EC. Scale bar=100 µm. **D** EdU-positive cells were quantified as a percentage of total EC number (n=4). **E** Immunostaining of p21 (red) and DAPI (blue) in cultured EC. **F** Quantification of p21 nuclear fluorescence intensity, relative to a female control (n=6; a.u.=arbitrary units). Mitochondrial, glycolytic and total ATP production rates were assessed using Seahorse, adjusting for cell density per well (n=4). **J** EC stained with MitoBrilliant (red) and DAPI (blue) Scale bar=100 µm. **K** Quantification of mitochondrial lengths (n=10). Data points reflect independent experiments and are plotted to show individual experimental results. Data in B,D,G-I were analyzed using multiple t-tests comparing 1-d and 6-d to control, adjusting for multiple comparisons using Holm-Sidak. Data in F and K were analyzed by 2-way ANOVA (Treatment x Sex) with Sidak post-hoc tests when main effects were significant. *P<0.05; **P<0.01; ***P<0.001

We hypothesized that the suppression of female EC proliferation and other transcriptomic signatures observed in vivo reflect a consequence of long-term loss of FoxO1 function rather than an immediate effect. To investigate this in greater detail, we utilized cultured adipose EC subjected to varying durations of FoxO1 inhibition. Cells were exposed to the FoxO1 inhibitor AS1842856 either continuously for 6 days (6-d, long-term inhibition) or only during the final 24 h of a 6-day culture period (1-d, short-term inhibition) and were compared with vehicle-treated cells maintained in growth media for 6-d (Fig. 5A). Consistent with the established role of FoxO1 as a suppressor of proliferation, short-term FoxO1 inhibition (1-d) increased EC proliferation in both sexes, evidenced by increased *Mki67* expression and higher EdU incorporation (Fig. 5B–D). In contrast, prolonged (6-d) FoxO1 inhibition produced a sex-distinct effect on endothelial proliferation, primarily affecting the fraction of cells entering S phase. *Mki67* transcript levels in both sexes did not differ from control following 6-day FoxO1 inhibition. Notably, EdU incorporation was significantly suppressed in female EC relative to controls but remained elevated in male EC (albeit at a reduced magnitude compared with 1-d inhibition) (Fig. 5B-D). Importantly, the anti-proliferative effect of FoxO1 inhibition in sub-confluent female EC emerged as early as 3 days after inhibition (Fig. S5A). Concomitant with reduced S-phase entry, sustained FoxO1 inhibition selectively induced nuclear accumulation of the cell cycle inhibitor p21^Cip1^ in female EC (Fig. 5E, F). Given that transcriptomic analyses revealed increased *Tgfb1* mRNA in FoxO1-deficient female EC, and that p21 can be induced by TGFβ1 signaling [29], we tested whether TGFβ1 could contribute to the anti-proliferative effect of FoxO1 inhibition. Consistent with the in vivo transcriptomic data, siRNA-mediated FoxO1 knockdown selectively increased *Tgfb1* mRNA expression in cultured female EC (Fig. S5B, C). Accordingly, nuclear levels of pSmad2, the transcriptional effector of Alk5-dependent TGFβ signaling, were increased in female EC following 6-d FoxO1 inhibition (Fig. S5D, E). However, pharmacological inhibition of TGFβ signaling using the TGFβR2 inhibitor RepSox (5 μM) further repressed, rather than rescued, proliferation in FoxO1-inhibited cells (Fig. S5F). Altogether, these findings indicate that the acute pro-proliferative response to FoxO1 inhibition is transient and not sustained with prolonged inhibition in either sex, highlighting an important temporal context to the effects of FoxO1 inhibition. Furthermore, the selectively suppression of S-phase entry in female EC following 3- or 6-d FoxO1 inhibition challenges the prevailing view of FoxO1 as solely a gatekeeper of EC quiescence and reveals a context-dependent, sex-specific regulation of endothelial proliferation mediated by a p21-dependent, TGFβ-independent mechanism.

### FoxO1 inhibition selectively impairs mitochondrial energy production and promotes oxidative stress in female endothelial cells

As proliferation is an energy-intensive process that requires coordinated increases in metabolic flux to sustain cell cycle progression, and TGFβ pathway activation did not account for the anti-proliferative effects of prolonged FoxO1 inhibition in female EC, we considered whether metabolic dysfunction could underlie this phenotype. Seahorse metabolic assays were utilized to assess real-time cellular ATP production via monitoring of oxygen consumption and extracellular acidification rates (Fig. S6A-D). Notably, total ATP production increased in male, but not female, EC following 1-d FoxO1 inhibition (Fig. 5G), despite increased proliferation in both cell types with this treatment regime. Pathway-resolved analysis of ATP production indicated increased glycolytic ATP generation in both sexes following 1-d FoxO1 inhibition (Fig. 5H), consistent with prior reports [21, 30]. In contrast, mitochondrial ATP production was selectively reduced in female EC following 1-d FoxO1 inhibition (Fig. 5I), indicating a sex-specific vulnerability of mitochondrial metabolism to FoxO1 inhibition. Following prolonged (6-d) FoxO1 inhibition, both total and mitochondrial ATP production were impaired in female EC (Fig. 5G,I). In contrast, total, glycolytic and mitochondrial ATP production at this time point were not different from control in male EC (Fig. 5G–I), indicating that sustained FoxO1 inhibition exerted minimal long-term impact on metabolic pathways in male EC.

Given that transcriptomic analyses had revealed significant downregulation of gene sets associated with mitochondrial function (Fig. 4D) in adipose EC from FoxO1 KD females, we tested whether the compromised mitochondrial ATP production in female EC following FoxO1 inhibition reflected transcriptional repression of mitochondrial machinery. However, transcripts of genes encoding multiple mitochondrial electron transport chain components (*Cox7a1, Cox7c, CytB*) were increased in EC of both sexes following 6-day FoxO1 inhibition (Fig. S6F-H). Because mitochondrial function critically depends on the integrity of mitochondrial network organization [31], we examined the impact of FoxO1 inhibition on mitochondria morphology in male and female adipose EC (Fig. 5J, K and Fig. S6E). In control cells of both sexes, mitochondria exhibited elongated, fused networks. After 1-d FoxO1 inhibition, mitochondrial architecture appeared similar to control. However, after 6-d FoxO1 inhibition, mitochondrial organization was selectively disrupted in female EC, characterized by diffuse staining and reduced mitochondrial network length. Conversely, mitochondrial network length increased in male EC following 6-d FoxO1 inhibition (Fig. 5J, K) Because FoxO1 has been implicated in regulation of mitochondrial dynamics in hepatocytes [32], we assessed levels of several genes encoding factors that control mitochondrial fusion and fission. However, mRNA levels of *Mfn2* and *Fis1* were not altered in cultured EC with prolonged FoxO1 inhibition (Fig. S6I,J), suggesting control of these events is post-transcriptional. Together, these data reinforce the idea that FoxO1 inhibition elicits sexually divergent effects on mitochondrial integrity, with female EC susceptible to mitochondrial disorganization following loss of FoxO1 function.

### FoxO1 inhibition induces oxidative stress and DNA damage selectively in female EC

**Fig. 6:**
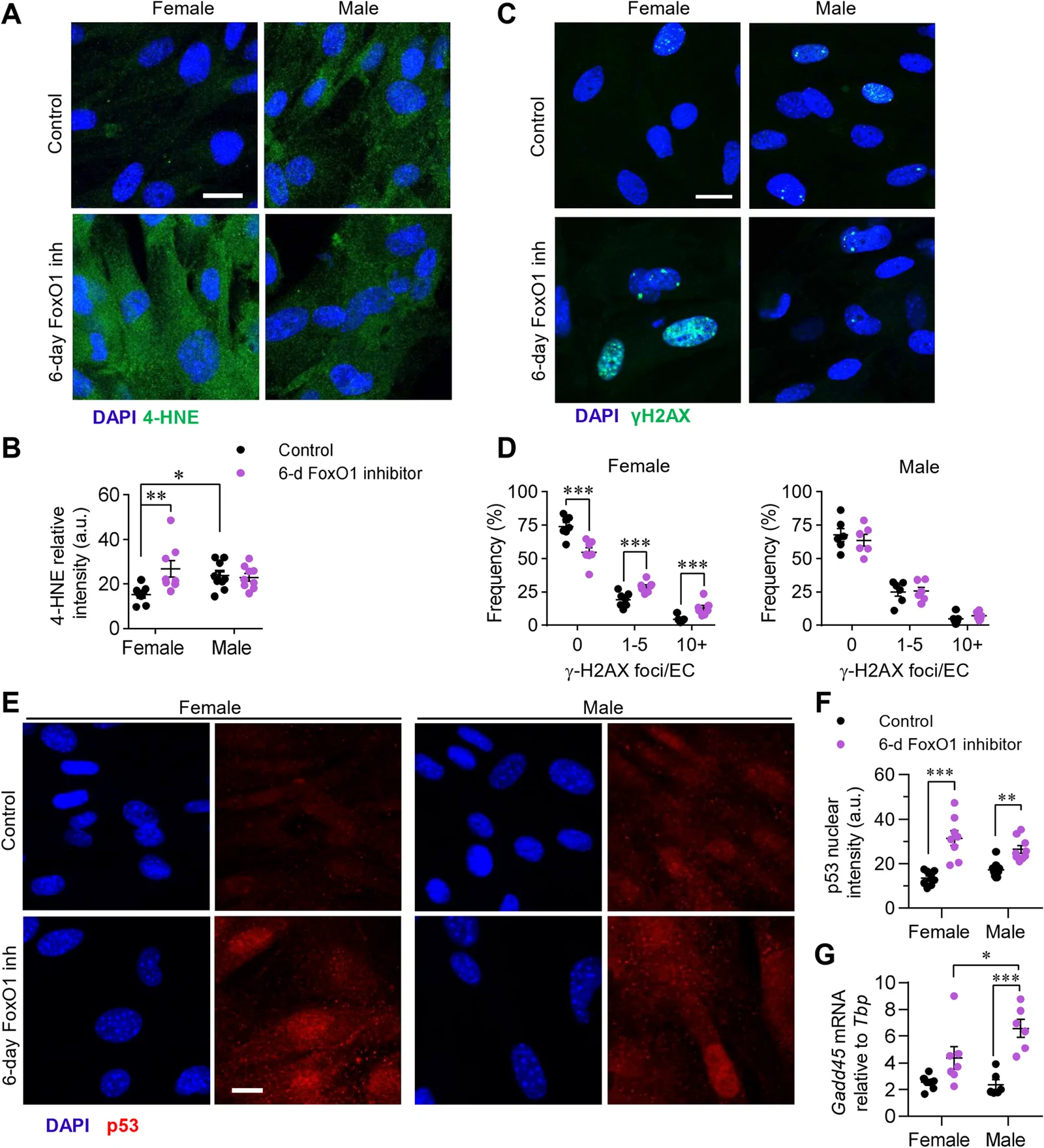
FoxO1 inhibition increases oxidative stress and DNA damage only in female EC. Female and male EC were cultured for 6 days with vehicle (Control) or FoxO1 inhibitor (AS1842856, 1 µM). **A** 4-HNE (green) immunostaining and DAPI (blue) to label nuclei. **B** Quantification of relative intensity of 4-HNE staining (n=8–9). **C** γH2AX (green) immunostaining and DAPI (blue). **D** Quantification of γH2AX foci per EC (n=6–7). **E** Immunostaining for p53 (red) and DAPI (blue). **F** Relative intensity of p53 nuclear fluorescence intensity (n=8–9/condition). **G** mRNA level of *Gadd45* following FoxO1 inhibitor treatment, normalized to housekeeping *Tbp* (2^-ΔCT) (Female n=7, Male n=6). Scale bars in A, C, E=20 µm. Data points reflect independent experiments and are plotted to show individual experimental results. Data were analyzed using 2-way ANOVA (Treatment x Sex) with Sidak post-hoc tests when main effects were significant *P<0.05; **P<0.01; ***P<0.001

Mitochondrial dysfunction is a major source of excessive reactive oxygen species (ROS), leading to cellular oxidative stress [31]. Consistent with this notion, transcriptomic analysis revealed significant enrichment of superoxide metabolism-related genes in adipose EC from female FoxO1 KD mice (Fig. 4D). In cultured cells, we quantified 4-hydroxy-2-nonenal (4HNE)-protein adducts, a product of lipid peroxidation, as an indicator of accumulated oxidative stress. Under control conditions, female EC exhibited lower levels of 4HNE compared to male cells (Fig. 6A, B), prolonged FoxO1 inhibition resulted in a significant accumulation of 4HNE in female but not male EC (Fig. 6A, B). Of note, 4HNE levels in FoxO1-inhibited female EC reached those observed in male EC, indicating loss of the female-specific redox advantage upon FoxO1 inhibition. This selective increase in oxidative stress occurred despite upregulation of the mitochondrial antioxidant genes *Prdx3* and *Gpx8,* which were similarly induced in EC from both sexes after 6-d FoxO1 inhibition (Fig. S6K, L).

Oxidative stress is a well-established driver of DNA damage and can simultaneously impair DNA repair pathways [33]. Given that base excision repair and mismatch repair ontologies were selectively downregulated in the transcriptome of female EC from EC-FoxO1 KD mice (Fig. 4D), we next examined DNA damage accumulation in cultured EC following 6-d FoxO1 inhibition. Immunostaining for phosphorylated histone H2AX (γH2AX), a marker of DNA double-strand breaks [34], showed a significant increase in the number of γH2AX foci per cell exclusively in female EC after 6-d FoxO1 inhibition. This increase was characterized by a higher proportion of female EC displaying 1–5 and >10 γH2AX foci (Fig. 6C, D), indicative of an increased burden of DNA damage. Although nuclear levels of p53, a transcriptional regulator of DNA repair processes [35], were comparably elevated in both male and female ECs after 6-d FoxO1 inhibition (Fig. 6E, F), expression of *Gadd45a*, a canonical p53 and FoxO1 target involved in DNA repair and maintenance of genomic stability [36], was selectively induced in male EC following FoxO1 inhibition (Fig. 6G).

Altogether, these data indicate that the increased DNA damage burden observed in female EC following prolonged FoxO1 inhibition arises from the combined effects of elevated oxidative stress and an attenuated DNA damage repair response.

### Prolonged FoxO1 inhibition induces a ROS-dependent senescence-like phenotype selectively in female endothelial cells

**Fig. 7:**
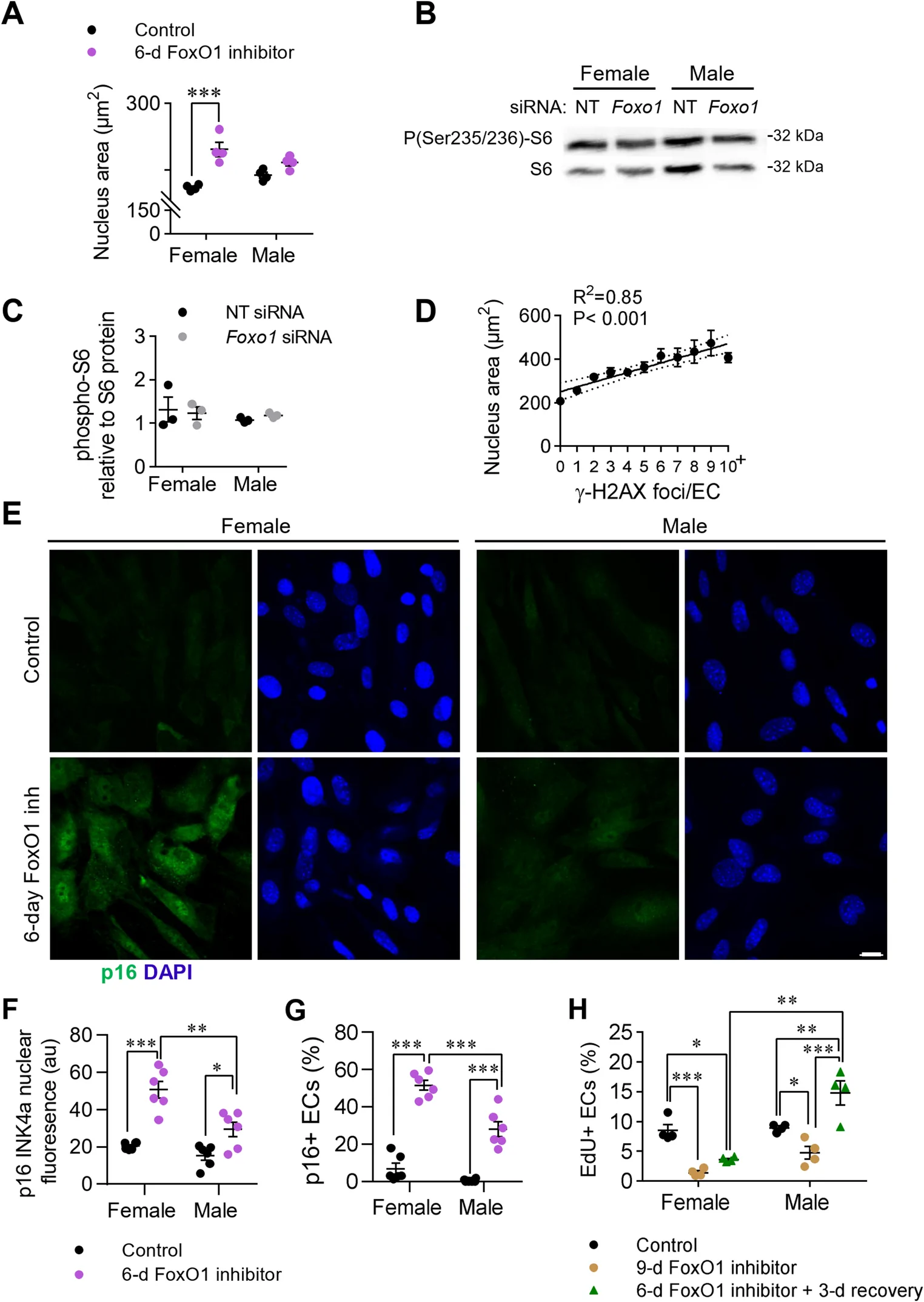
FoxO1 inhibition induces a pro-senescent phenotype in female EC. **A** The effect of 6-d FoxO1 inhibitor on nuclear area of cultured EC. **B** Western blot of cultured adipose EC treated for 3-d with non-targeting (NT) or Foxo1 siRNA. **C** Protein quantification of phosphorylated (Ser235/236) relative to total S6 ribosomal protein (n=3). **D** Correlation between number of γH2Ax foci and nuclear area across all conditions (n=12). **E** p16INK4a immunostaining co-stained with DAPI. Scale bar=20 µm. **F** p16INK4a fluorescence intensity quantified relative to a female control (n=6). **G** % p16INK4a-positive cells in female and male EC (n=6). **H** EdU incorporation in cultured EC following 9-d FoxO1 inhibition or 6-d inhibition with 3-d recovery. Each data point reflects independent experiments, plotted with the group mean ± SEM. Data were analyzed using 2-way ANOVA (Treatment x Sex) with Sidak post-hoc tests when main effects were significant *P<0.05; **P<0.01; ***P<0.001

**Fig. 8:**
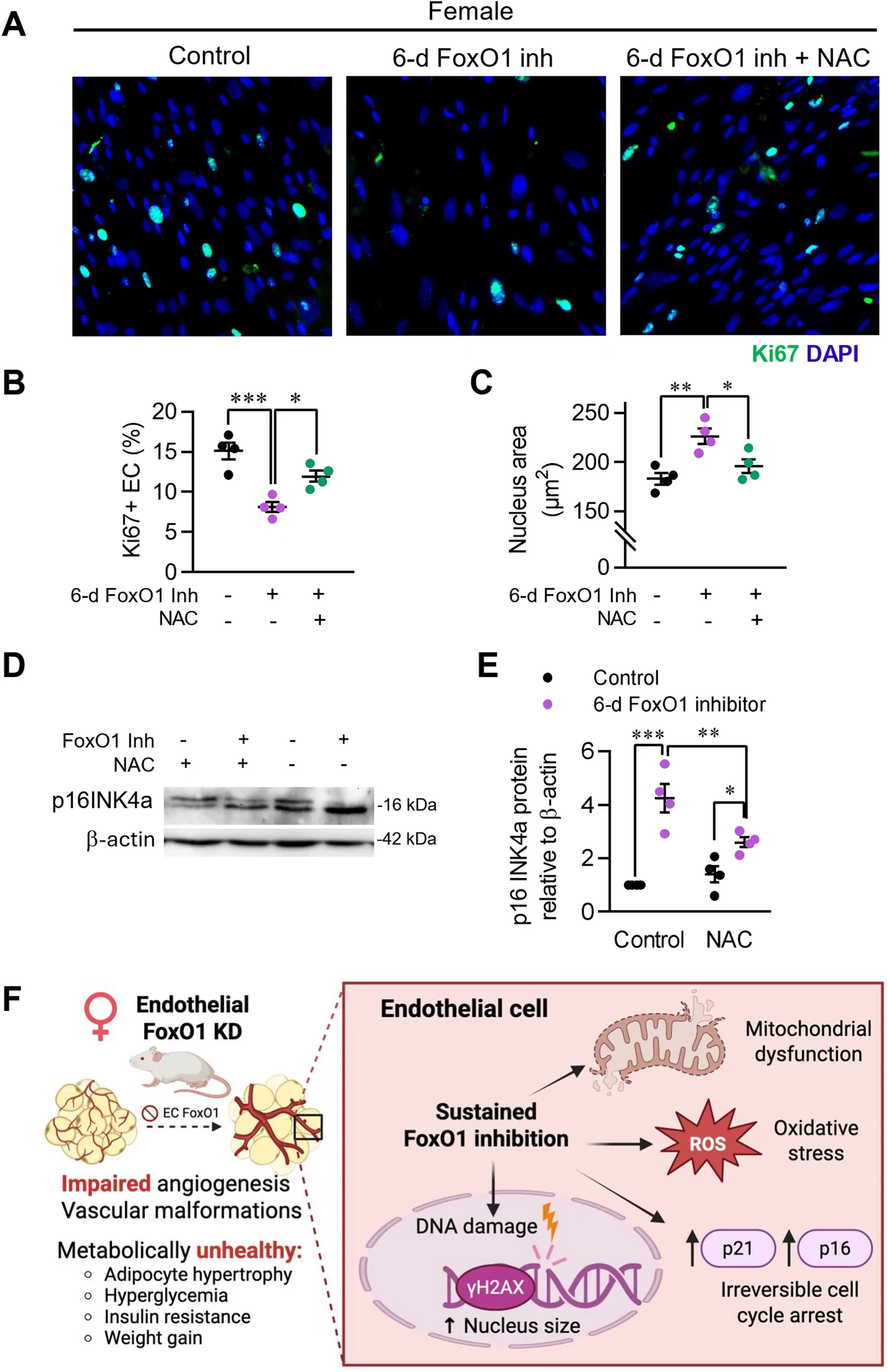
Antioxidant treatment of female EC reverses the anti-proliferative, pro-senescent phenotype caused by FoxO1 inhibition. Female EC were treated for 6-days with vehicle, FoxO1 inhibitor (1 µM) or FoxO1 inhibitor and NAC (2.5 mM) (n=4). **A** Cells stained with Ki67 antibody and DAPI. **B** Quantification of Ki67+ EC as a percentage of total EC number. **C** Nuclear size was assessed based on DAPI stain. **D** p16INK4a and housekeeping protein β-actin were detected by Western blot. **E** Protein quantification of p16INK4a relative to β-actin. **F** Summary schematic illustrating key influences of FoxO1 knock down or inhibition in female EC. Data points reflect independent experiments and are plotted with the group mean ± SEM. Data were analyzed by 1-way ANOVA **B**, **C** or 2-way ANOVA **E** with Sidak post-hoc tests when main effects were significant *P<0.05; **P<0.01; ***P<0.001. Schematic created in BioRender. Rudnicki, M. (2026) https://BioRender.com/h25glu7

Persistent, unresolved DNA damage is a well-established trigger of cellular senescence. One hallmark of senescence is increased cell size. Consistent with this, nuclear size, a proxy for overall cell size [37] was selectively increased in female EC following 6-days of FoxO1 inhibition (Fig. 7A). This enlargement was not attributable to increased protein synthesis, as indicated by unchanged phosphorylation of ribosomal protein S6 (Fig. 7B, C). Notably, nuclear enlargement and accrual of γH2AX was detectable in female EC after shorter durations of FoxO1 disruption (3-days FoxO1 siRNA-mediated knockdown or 3-days of FoxO1 inhibition) (Fig. S7A-G), and was positively correlated with the number of γH2AX foci per cell (Fig. 7D), directly linking nuclear expansion to DNA damage burden. In parallel, nuclear levels of the pro-senescence marker p16^INK4a^ increased to a greater extent, and a greater percentage of cells were positive for p16^INK4a^, in female EC following 6-d of FoxO1 inhibition (Fig. 7E–G). Because senescence is defined by irreversible cell cycle arrest, we next tested whether EC proliferation could recover following removal of the FoxO1 inhibitor. Prolonged FoxO1 inhibition for 9 days suppressed proliferation not only in female but also male EC. Notably, only male EC recovered their proliferative capacity 3-d after removal of FoxO1 inhibition, whereas the proliferative rate of female EC remained significantly lower than control (Fig. 7H). Given the elevated oxidative stress observed in female EC, we next tested whether excess ROS acts upstream to drive the senescent phenotype [38]. To directly test this possibility, we co-treated female EC with the general antioxidant N-acetylcysteine (NAC) during FoxO1 inhibitor exposure. NAC treatment significantly improved proliferative capacity relative to female EC treated with the FoxO1 inhibitor alone (Fig. 8A, B). Similarly, NAC prevented nuclear enlargement (Fig. 8C) and significantly lowered p16^INK4a^ protein levels in the FoxO1 inhibitor-treated female EC (Fig. 8D, E). Collectively, these findings demonstrate that loss or inhibition of FoxO1 compromises endothelial resilience and promotes a senescence-like state in female EC and further indicate that ROS scavenging can mitigate the adverse consequences of sustained FoxO1 inhibition.

## Discussion

Under obesogenic conditions, female mice exhibit enhanced adipose angiogenesis and superior EC proliferative capacity compared to males [9, 18]. Here we identify FoxO1 as a critical determinant of this sexual dimorphism, demonstrating that it preserves fundamental EC cellular processes in females, thereby contributing to vascular homeostasis and limiting female susceptibility to the metabolic complications of diet-induced obesity. This conclusion is supported by converging evidence showing that FoxO1 expression and activity are higher in female EC and that its loss in female EC led to impaired angiogenesis, aberrant vascular remodeling, and metabolic dysfunction that are exacerbated by high-fat diet (Fig. 8F). At the cellular level, sustained genetic loss of FoxO1 induces a pathological reprograming of the female EC transcriptomic signature. Key features of this dysfunctional phenotype, including cell cycle arrest, mitochondrial dysfunction, oxidative stress, DNA damage and a senescence-like phenotype, were also observed in cultured female adipose EC following sustained pharmacological inhibition of FoxO1. Together, our findings highlight FoxO1 as an essential resilience factor in female EC, which underpins their resistance to metabolic stressors.

FoxO1 has been described as an enforcer of endothelial quiescence and a regulator of vascular homeostasis [21]. We and others have previously shown that short-term FoxO1 inhibition promotes proliferation and glycolysis of endothelial cells [9, 20, 21, 28, 39–41]. However, G1 arrest has been reported in human umbilical vein EC that were assessed more than one week following lentiviral shRNA-mediated knockdown of FoxO1 [19]. Our findings reconcile these opposing observations by demonstrating that FoxO1 exerts time-dependent effects on endothelial proliferation**,** with transient inhibition promoting cell cycle entry whereas sustained inhibition provokes cell cycle arrest. We propose that sustained FoxO1 loss or inhibition drives proliferative arrest through cumulative metabolic stress, oxidative damage, and replicative exhaustion following an initial proliferative burst. In line with this hypothesis, sustained FoxO1 inhibition was associated with increased nuclear p53 levels, indicative of a stress response that may contribute to cell cycle arrest [42].

The pronounced effects of sustained FoxO1 inhibition in female EC reveal a heightened, intrinsic reliance on FoxO1. Elevated FoxO1 expression were observed in female adipose EC from both human and mouse samples. In addition, the consistently higher FoxO1 levels and transcriptional activity observed in female mouse adipose EC across multiple experimental conditions indicate that this sex difference is a stable, cell-autonomous feature. Beyond its canonical role in restraining proliferation, our data indicate that FoxO1 maintains mitochondrial integrity, redox balance, and genomic stability in female EC, suggesting integration into sex-specific transcriptional networks that prioritize stress resilience. Consistent with this, female EC possess a greater proliferative capacity than male EC, despite higher FoxO1 levels [18], and FoxO1 loss elicited a shift in the female EC transcriptome, characterized by down-regulation of genes associated with proliferation and nucleosome organization. Notably, FoxO1-deficient female EC adopt a transcriptional profile resembling that of EC from control male mice, which are more vulnerable to diet-induced vascular dysfunction [18]. Functionally, this transition was accompanied by impaired capillarization in vivo and a senescence-like phenotype in vitro, marked by mitochondrial disruption, oxidative stress and DNA damage. Importantly, although proliferation was reduced in both sexes, male EC did not exhibit the mitochondrial dysfunction, oxidative stress or senescence-like features observed in females and recovered proliferative capacity following inhibitor withdrawal, indicating a divergence in the consequences of FoxO1 loss beyond proliferation alone.

Mechanistically, our findings point to mitochondrial dysfunction and oxidative stress accumulation as drivers of the female-specific phenotype. Female EC exhibit higher mitochondrial content and oxidative metabolism than male EC [18, 43]. This fundamental sex difference, which has also been reported in other cell types and species [44, 45], may render female cells more susceptible to mitochondrial dysfunction. Indeed, we found that FoxO1 loss in female EC caused mitochondrial fragmentation, a structural change that has been reported to compromise energetic efficiency and increase superoxide generation [46–48]. Accordingly, FoxO1 inhibition in female EC led to elevated lipid peroxidation, accumulation of DNA damage, and reduced induction of DNA repair programs relative to males [49]. Co-treatment with the glutathione precursor NAC partially rescued proliferation and restored p16 levels in FoxO1 inhibitor-treated female EC, which implicates the antioxidant buffering capacity of cells as a factor that determines the cellular response to FoxO1 inhibition. Thus, our data support the concept that mitochondrial disruption, oxidative stress and unresolved DNA damage, well-established drivers of cellular senescence [38, 49, 50], are key drivers of the female EC dysfunction following FoxO1 loss or inhibition. Although these events were functionally validated in cultured EC, direct in vivo assessment of the effects of FoxO1 loss on endothelial mitochondrial morphology and oxidative stress in adipose tissue will be valuable to pursue in future studies.

Moreover, our findings are consistent broadly with literature linking female sex with longevity and greater resistance to aging-associated senescence [51]. A recent study reported that female human umbilical vein EC were resistant to replicative stress-induced senescence, compared to male counterparts [52]. Additionally, extensive investigations have established the FoxO family of transcription factors are established “longevity factors”. Notably, our data link these concepts by highlighting a central role for FoxO1 as a mediator of female EC resilience.

The considerable functional impairments induced by sustained FoxO1 inhibition in female EC at the cellular level provide insight into plausible mechanisms underlying the defective angiogenesis of the adipose capillary network as well as reduced vascular density in other metabolic organs (brown adipose tissue and skeletal muscle) in female EC-FoxO1 KD mice. Beyond highlighting sex-specific effects of endothelial FoxO1, our findings reinforce the growing body of evidence that impaired vascularization of adipose and skeletal muscle tissues contributes causally to obesity-related metabolic dysfunction. Indeed, the reduced capillarization detected following EC-FoxO1 KD in female mice was accompanied by increased adiposity and impaired glucose homeostasis. These deleterious outcomes occurred only in female mice despite substantial increases in capillary diameters in both sexes. The opposite was seen with EC-FoxO1 knockdown in male mice, wherein enhanced tissue capillarization and improved metabolic outcomes exemplified the benefit of improving adipose vascularization [9]. These findings align well with the core concept that vascularization is a key determinant of peripheral tissue function and systemic metabolic homeostasis. Additionally, the excessive capillary enlargement in the female EC-FoxO1 KD mice may compromise nutrient and oxygen transfer [53], further contributing to tissue dysfunction. Lastly, we cannot rule out the possibility that effects detected in EC-FoxO1 KD females may result uniquely from the senescence-like state of the EC in these mice. Indeed, a causative role of EC senescence in impaired adipose and whole-body tissue homeostasis was recently shown, which was suggested to be independent of vascular density [54].

In summary, our study uncovers a sex-biased requirement for FoxO1 in EC biology. In contrast to previous studies describing FoxO1 primarily as a suppressor of endothelial proliferation, our data provide evidence that FoxO1 serves as a sex-specific regulator of endothelial stress resilience, revealing a previously unrecognized divergence in FoxO1 signaling between male and female endothelium. While the basis for elevated FoxO1 expression in female EC remains to be established, our data illustrate the exacerbated consequences of FoxO1 loss in females both in vivo and in cultured EC independent of hormonal cues. Female EC display a heightened, cell-intrinsic dependence on FoxO1 to sustain fundamental cellular functions, such as mitochondrial integrity, genomic stability and resistance to senescence. Consequently, loss of endothelial FoxO1 in females compromises cellular resilience, impairs angiogenesis and disrupts vascular architecture, culminating in adverse metabolic outcomes. These observations support a model in which FoxO1 acts as a sex-specific regulator of EC resilience, vascular health and systemic metabolic homeostasis. These insights may have broader implications for understanding sex differences in vascular aging and disease susceptibility.

## Materials and methods

### Animals

All animal procedures were conducted in accordance with protocols approved by the York University Animal Care Committee. Mice were housed under specific-pathogen-free conditions in ventilated cages and received water and standard chow diet ad libitum until initiation of experiments. Young adult wild-type mice (FVB/NJ background) fed a standard chow were used for adipose EC isolation to initiate cell cultures.

A colony of conditional *Foxo1* mice crossed with transgenic mice expressing tamoxifen-inducible Cre recombinase (CreERT2) under the control of the endothelial-specific Pdgfb promoter (*Pdgfb*-*iCreER*^*T2*^*;Foxo1*^*f/f*^) [9], were used for analyses of the tissue-level and whole-body effects of FoxO1 deletion. This colony was maintained on an FVB/NJ background for >10 generations. Endothelial specificity of *Pdgfb*-driven Cre expression was validated by crossing *Pdgfb*-*iCreER*^*T2*^*;Foxo1*^*f/f*^ with the double-fluorescent Cre reporter mouse *Rosa*^*mT/mG*^ (JAX stock # 007676). To induce Cre-mediated recombination, mice (6 weeks of age) received intraperitoneal injections of tamoxifen (200 μL *i.p.*; 15 mg/mL in corn oil, Sigma-Aldrich) for five consecutive days, as described previously [9, 18]. In all experiments, age-matched tamoxifen-injected *Foxo1*^*f/f*^ littermates lacking Cre were used as controls.

### Mouse diet-induced obesity model

One week following the final tamoxifen injection, female endothelial-specific FoxO1 knockdown mice (EC-FoxO1 KD) and control littermates were placed on either a low-fat (LFD; 11 kcal% fat w/sucrose, Research Diets D12329) or a high-fat diet (HFD; 58 kcal% fat w/sucrose Surwit Diet, Research Diets D12331) for 4, 7 or 16 weeks. This HFD intervention was previously found to induce obesity and disrupt systemic glucose homeostasis and lipid handling in male mice [9, 18]. A cohort of male littermates received similar diets for equivalent durations for sex comparisons. Diet allocation was based on genotype and age-matching of the available mice. Researchers were not blinded to sex, genotype or diet. N values for specific assessments are described in each figure legend. For assessment of EC proliferation and transcriptomic analyses, a separate cohort of EC-FoxO1 KD mice (n = 5 females, n = 6 males) was fed the HFD for 7 weeks and injected with EdU (1.5 mg in 100 μL sterile PBS; *i.p.*) on two consecutive days prior to tissue collection. Mice were deeply anesthetized using inhaled isoflurane prior to tissue extraction and euthanized by exsanguination while fully anesthetized.

### Intra-peritoneal insulin and glucose tolerance tests (ITT and GTT)

As indicators of glucose homeostasis, insulin and glucose tolerance tests were conducted on female LFD and HFD mice, at diet weeks 14 and 15, respectively. For ITT, mice were fasted for 4-h prior to receiving a bolus injection of insulin (1.0 U/kg BW, *i.p.*). For GTT, mice were fasted for 16-h prior to receiving a bolus injection of glucose (2 g/kg BW, *i.p.*). Blood glucose was measured by glucometer at post-injection time-points 0, 20 and 40 min (ITT) or 0, 30, 60, 90 and 120 min (GTT).

### Adipose tissue histochemistry

Small pieces of perigonadal white adipose tissue (pWAT) and brown adipose tissue (BAT) were fixed (4% paraformaldehyde) and processed for either whole-mount staining or paraffin embedding. For EC proliferation assessment, EdU+ cells were detected using a click reaction (0.1 nM Azide-fluor 545; 0.1 M Tris, 1 mM CuSO4, 0.1 M ascorbic acid) prior to immunostaining, as described previously [18], followed by PBS washes, then blocking and permeabilization with 0.5% Triton X-100 (PBS-T) and 5% goat serum at RT for 1 h. Samples were incubated overnight at 4 °C with primary anti-ERG antibody (1:100; Abcam ab133264) in blocking buffer. Following washes, samples were incubated with fluorescein-labeled *G. simplicifolia* lectin (1:50, GSL I–BSL I; FL-1101, Vector Labs) and the goat anti-rabbit Alexa 647 secondary antibody (1:200, #111–605-003, Jackson Immunoresearch) in blocking buffer for two hours. Samples were counter-stained with DAPI (1:1000) for 5 min, washed with PBS then mounted on a slide with VectaShield or Vibrance mounting media (#VECTH190010 or VECTH170010, Vector Labs). Single plane images were captured with a Zeiss LSM700 inverted confocal microscope equipped with a 40x/1.30 Oil EC PLAN Neofluar objective (York University YSciCore). Microvascular content, vessel diameter, EC nuclei and EdU positive nuclei were quantified from 4–5 fields of view per animal using ImageJ, as previously described [55]. For morphometric analysis of adipocytes, fixed WAT was paraffin embedded, sectioned and stained with hematoxylin and eosin by The Centre for Phenogenomics (Toronto, Canada). Adipocyte areas were analyzed in 4–6 randomly selected fields of view per mouse (10× objective) using ImageJ. For quantification of capillaries, de-paraffinized sections were stained with *G. simplicifolia* lectin-FITC (1:50) and Wheat Germ Agglutinin-Rhodamine (1:200; RL-1022, Vector Labs) and images captured using a Zeiss 200 M inverted microscope, cooled CCD camera and Metamorph software. Capillary and adipocyte counts were obtained from 3–4 independent fields of view per mouse (10X objective).

### Skeletal muscle histochemistry

Extensor digitorum longus (EDL) muscles were embedded in optimal cutting temperature (OTC) compound and frozen in liquid nitrogen-cooled isopentane. Transverse 10 µm cryosections of EDL muscles were co-stained with *G. simplicifolia* lectin FITC (1:100) and wheat germ agglutinin-Rhodamine (1:400) to visualize myofiber membranes. A Zeiss inverted microscope with a cooled CCD camera and Metamorph software was used to capture three to four representative images of each muscle section using a 20X objective. Capillary to fiber ratio (C:F) was measured by counting the number of capillaries relative to the number of myofibers in each field of view.

### RNA-sequencing

Perigonadal and inguinal subcutaneous white adipose depots from each mouse were harvested, pooled and digested with type I collagenase for 30 min at 37 °C as described previously [9, 18]. EC were purified from the filtered stromal vascular fraction by magnetic sorting following incubation with Dynabeads conjugated to rat anti-mouse VE-cadherin antibody and streptavidin-magnetic beads coupled to biotinylated rat anti-mouse CD31 antibody. RNA was extracted from freshly isolated EC using RNeasy Mini Kit and quality-checked using a Bioanalyzer automated electrophoresis system (Agilent Technologies Inc.). Samples with an RNA Integrity Number (RIN) > 6 (n = 4/group) were processed for RNA sequencing by The Centre for Applied Genomics, The Hospital of Sick Children, Toronto. Ribosomal RNA-depleted cDNA-libraries were generated and sequenced using a Novaseq SP flowcell (paired end 2×50 bp), generating ~60 million reads per sample. After mapping reads to the mouse genome from Ensembl Mus_musculus.GRCm38.cdna.all.fa, expression levels were normalized as Transcripts Per Kilobase Per Million (TPKM) by dividing the read count of each transcript model with its length and scaling the total per sample to one million using Kalisto V0.4.4. Quality control of raw and mapped reads was done using FastQC version 0.11.8. Transcripts with <20 raw counts per library were deemed unexpressed and were discarded from analysis. Data for control mice were previously used to analyze male–female differences in EC transcriptomes (GEO GSE216585) [18]. Here, differential expression analysis was performed comparing control and EC-FoxO1 KD mice, using the R package EdgeR version 3.34 with the formula log2CPM = 0 + +genotype between control and EC-FoxO1 KD for each sex, correcting for common, trended and tagwise disparity. The analysis rendered two-base logarithmic fold change (log2FC) differences and false discovery rate (FDR) values, where FDR of <5% was considered significant.

### Bioinformatics analyses

Principal components were calculated to reduce dimensionality using only the DEGs identified in female samples. Gene expression values were normalized and scaled prior to analysis. Principal component analysis (PCA) was conducted based on gene expression profiles, using pcaMethods R package version 1.88.0. A PCA plot was generated to visualize the clustering and separation of samples based on the first two principal components (PC1 and PC2), which accounted for the greatest variance. Samples were compared using 2-way ANOVA based on PC1 distribution (which accounted for >50% of the variance). Gene set enrichment analysis (GSEA)[56] was used to test for enrichment of ontologies from publicly available databases Gene Ontology Biological Process and Wiki Pathways, comparing EC-FoxO1 KD to Control for each sex. Custom ‘EC proliferation’ and ‘EC SASP’ genesets were formulated based on literature, as described previously [18]. The logFC of the core enrichment genes from selected female ontologies were compared between males and females using a Wilcoxon test, reporting the FDR q-value.

### Bioinformatic analysis of human adipose EC gene expression

*FOXO1* expression was analysed using a recently published comprehensive single-cell atlas of the human subcutaneous adipose tissue vascular niche, comprising 68,503 vascular cells from 65 donors [23]. Feature and violin plots were generated in Seurat [57] (v5.3.0). Differential *FOXO1* expression between subECs from non-obese male and female donors was assessed using the Wilcoxon rank-sum test.

### Endothelial cell isolation for culturing

Pooled pWAT and sWAT depots of wild-type male and female FVB/N mice (fed standard diet) were digested for 30 min at 37 °C with type I collagenase and cells from the stromal vascular fraction were seeded in 1.5% gelatin-coated 25 cm^2^ flasks in high-glucose (25 mmol/L) DMEM supplemented with 20% FBS. After reaching confluence, cells were purified with streptavidin-magnetic beads coupled to biotinylated rat anti-mouse CD31 antibody. Cells were maintained in culture and used for experiments between passages 3 to 5.

### Cell culture experiments

Cells were plated in multi-well plates for proliferation assays, RNA or protein, or 8-well chamber slides coated with rat tail collagen for immunostaining. For luciferase-reporter assays, cells were transfected with pGL3-basic (#E1751, Promega), FRE [forkhead response element; RRID:Addgene_1789; originating from the lab of Michael Greenberg (Boston University)] subcloned into pGL3, and pRL (Renilla luciferase; #E2231, Promega) using Lipofectamine 3000 (#L3000001; Invitrogen) and luciferase activity was assessed 24 h later, using the Dual Luciferase Assay Reagent kit (#PRE1910, Promega). For siRNA treatments, cells were plated overnight, then transfected with SMARTpool non-targeting or FoxO1 siRNA (5 nM) using DharmaFECT 1 transfection reagent; #T-2001-01, Horizon Discovery) then cultured for 24–72 h prior to analysis. Cells were serum-starved for 2 h prior to lysis. To inhibit FoxO1 function, cells were treated with the FoxO1 inhibitor AS1842856 (1 µM; #344,355, EMD Millipore) for 1, 3, 6 or 9 days. In some experiments, cells were concurrently treated with N-acetylcysteine (NAC, 2.5 mM; #A9165, Sigma-Aldrich), or the TGFBR (Alk5) inhibitor RepSox (5 µM; HY-13012; Tocris). Media was replenished every three days with the specified reagents. To test recovery from FoxO1 inhibition, EC were cultured for 9 days with vehicle, with FoxO1 inhibitor, or with FoxO1 inhibitor for 6 days followed by 3 days with vehicle only. For proliferation assays, cells were treated with EdU (10 µM for 4 h) prior to fixation of cells with 4% paraformaldehyde. In some experiments, cells were incubated with MitoBrilliant™ (100 nM; #7700; Tocris) for 1 h prior to fixation.

### Cell immunostaining

Following fixation with 4% paraformaldehyde, non-specific binding was blocked with 0.1% Triton and 5% goat serum in PBS for one hour, then cells were incubated overnight at 4 °C with primary antibodies diluted in blocking buffer: Cells then were incubated for one hour with secondary antibodies diluted in blocking solution. See Table S3 for antibody details. Cells were incubated with DAPI (1:1000) to detect the nuclei, washed with PBS and cover-slipped using VectaShield antifade mounting media. Images were captured using a Zeiss LSM700 or a Zeiss Observer Z1 SD confocal (40× objective), maintaining identical gain settings within each experiment. The average pixel intensity per nucleus (p16, p21, p53, pSmad2) or per cell area (4-HNE) was measured from 5 to 8 fields of view per experiment using ImageJ. DNA damage was assessed by counting γH2AX foci per nucleus. Mitochondrial network quantification was performed using Image J (Fiji). Average mitochondria lengths were calculated from thresholded 2D images using the Mitochondria Analyzer plugin [58].

### Metabolic assessments

Cultured endothelial cells were plated at a density of 15,000 cells/well in Agilent Seahorse 96-well plates and were either treated with FoxO1 inhibitor AS1842856 as previously described or left untreated. The Seahorse XF Real-Time ATP Rate Assay (#103,592–100, Agilent) was then conducted according to manufacturer’s instructions using the Seahorse XFe96 analyzer and Agilent Seahorse Wave software. Calculated glycoATP and mitoATP production rates, expressed as pmol ATP/min, were adjusted to cellular DNA content per well (CyQUANT #C7026, ThermoFisher).

### RNA isolation and qPCR

RNA was extracted from freshly isolated EC, cultured endothelial cells, pWAT and brown adipose tissue (BAT) using the RNeasy Mini Kit (Qiagen Inc.) or Trizol, followed by reverse transcription using M-MuLV reverse transcriptase. cDNA was analyzed by real-time PCR on the Rotor-Gene Q platform using TaqMan primer/probe sets (see Table S4 for details) and TaqMan Master Mix (#4444963, Invitrogen or #ABMG890, Diamed). Transcript levels of each target gene were calculated relative to the housekeeping gene and expressed as 2^−ΔCt^.

### Western blot

Total protein extraction from cultured adipose EC was performed as described previously using lysis buffer containing 50 mM Tris (pH 8.0), 150 mM sodium chloride, 1% Nonidet P-40, 0.5% sodium deoxycholate, 0.1% sodium dodecyl sulfate with phosphatase inhibitor and protease inhibitor. Following protein separation through a polyacrylamide gel under reducing conditions, western blots were performed using antibodies as specified in Table S3. Membranes were developed using enhanced chemiluminescence and densitometry analysis was performed with ImageJ Analysis Software (NIH).

### Statistics

For mouse studies, diet allocation was based on genotype and age-matching of the available mice. Researchers were not blinded to sex, genotype or diet. No statistical methods were used to predetermine sample size and the experiments were not randomized. Metabolic tests and tissue collections were conducted for all groups within the same time of day to avoid confounding influences associated with day/night cycle. Data are reported with each mouse considered as one independent biological replicate. No outliers were excluded from our analyses. Averaged values were used when technical replicates (analysis of the same sample) were performed. For in vitro assays, cells isolated from each mouse were maintained as independent biological samples. Experiments were repeated four to six times with independent biological samples. Averaged values were used when technical replicates (analysis of the same sample) were performed. Data were analyzed using Students t test, 1-way or 2-way ANOVA with post-hoc testing, where appropriate.

## Supplementary Information

Below is the link to the electronic supplementary material.

Supplementary Material 1

Supplementary Material 2

Supplementary Material 3

## Data Availability

The authors declare that the data supporting the findings of this study are available within the paper and its Supplementary Information files. Raw data files are available from the corresponding author upon reasonable request.

## References

[1] Choe SS, Huh JY, Hwang IJ et al (2016) Adipose tissue remodeling: its role in energy metabolism and metabolic disorders. Front Endocrinol 7:30. 10.3389/fendo.2016.00030 PMC4829583 27148161

[2] Maeyens LT, Nelson JF, Zhao S (2026) Visceral adiposity, metabolic health and aging. Nat Aging. 10.1038/s43587-026-01076-4 41714834

[3] Logue J, Walker JJ, Colhoun HM et al (2011) Do men develop type 2 diabetes at lower body mass indices than women? Diabetologia 54:3003–3006. 10.1007/s00125-011-2313-3 PMC4220585 21959958

[4] Steinarsson AO, Rawshani A, Gudbjörnsdottir S et al (2018) Short-term progression of cardiometabolic risk factors in relation to age at type 2 diabetes diagnosis: a longitudinal observational study of 100,606 individuals from the Swedish national diabetes register. Diabetologia 61:599–606. 10.1007/s00125-017-4532-8 PMC6448968 29318343

[5] Corvera S, Solivan-Rivera J, Yang Loureiro Z (2022) Angiogenesis in adipose tissue and obesity. Angiogenesis 25:439–453. 10.1007/s10456-022-09848-3 PMC9519636 35857195

[6] AlZaim I, de Rooij LPMH, Sheikh BN et al (2023) The evolving functions of the vasculature in regulating adipose tissue biology in health and obesity. Nat Rev Endocrinol 19:691–707. 10.1038/s41574-023-00893-6 37749386

[7] Lee Y, Tukei KL, Fang Y et al (2025) Obesity alters the vascular morphology and VEGF-A signaling in adipose tissue. FASEB Bioadv 7:e70018. 10.1096/fba.2025-00027 PMC12147507 40496350

[8] Shimizu I, Aprahamian T, Kikuchi R et al (2014) Vascular rarefaction mediates whitening of brown fat in obesity. J Clin Invest 124:2099–2112. 10.1172/JCI71643 PMC4001539 24713652

[9] Rudnicki M, Abdifarkosh G, Nwadozi E et al (2018) Endothelial-specific FoxO1 depletion prevents obesity-related disorders by increasing vascular metabolism and growth. Elife 7:e39780. 10.7554/eLife.39780 PMC6279348 30511639

[10] An YA, Sun K, Joffin N et al (2017) Angiopoietin-2 in white adipose tissue improves metabolic homeostasis through enhanced angiogenesis. Elife 6:e24071. 10.7554/eLife.24071 PMC5391203 28355132

[11] Hasan SS, John D, Rudnicki M et al (2025) Obesity drives depot-specific vascular remodeling in male white adipose tissue. Nat Commun 16:5392. 10.1038/s41467-025-60910-2 PMC12198381 40562785

[12] Rudnicki M, Abdifarkosh G, Rezvan O et al (2018) Female mice have higher angiogenesis in perigonadal adipose tissue than males in response to high-fat diet. Front Physiol. 10.3389/fphys.2018.01452 PMC6206240 30405427

[13] Bloom SI, Islam MT, Lesniewski LA, Donato AJ (2023) Mechanisms and consequences of endothelial cell senescence. Nat Rev Cardiol 20:38–51. 10.1038/s41569-022-00739-0 PMC10026597 35853997

[14] Pasut A, Lama E, Van Craenenbroeck AH et al (2025) Endothelial cell metabolism in cardiovascular physiology and disease. Nat Rev Cardiol 22:923–943. 10.1038/s41569-025-01162-x 40346347

[15] Ungvari Z, Tarantini S, Kiss T et al (2018) Endothelial dysfunction and angiogenesis impairment in the ageing vasculature. Nat Rev Cardiol 15:555–565. 10.1038/s41569-018-0030-z PMC6612360 29795441

[16] Lorenz M, Blaschke B, Benn A et al (2019) Sex-specific metabolic and functional differences in human umbilical vein endothelial cells from twin pairs. Atherosclerosis 291:99–106. 10.1016/j.atherosclerosis.2019.10.007 31706078

[17] Lorenz M, Witt E, Völker U et al (2022) Serum starvation induces sexual dimorphisms in secreted proteins of human umbilical vein endothelial cells (HUVECs) from twin pairs. Proteomics 22:e2100168. 10.1002/pmic.202100168 35357760

[18] Rudnicki M, Pislaru A, Rezvan O et al (2023) Transcriptomic profiling reveals sex-specific molecular signatures of adipose endothelial cells under obesogenic conditions. iScience 26:105811. 10.1016/j.isci.2022.105811 PMC9823135 36624843

[19] Dharaneeswaran H, Abid MdR, Yuan L et al (2014) FOXO1-mediated activation of Akt plays a critical role in vascular homeostasis. Circ Res 115:238–251. 10.1161/CIRCRESAHA.115.303227 PMC4961050 24874427

[20] Furuyama T, Kitayama K, Shimoda Y et al (2004) Abnormal angiogenesis in Foxo1 (Fkhr)-deficient mice. J Biol Chem 279:34741–34749. 10.1074/jbc.M314214200 15184386

[21] Wilhelm K, Happel K, Eelen G et al (2016) FOXO1 couples metabolic activity and growth state in the vascular endothelium. Nature 529:216–220. 10.1038/nature16498 PMC5380221 26735015

[22] Andrade J, Shi C, Costa ASH et al (2021) Control of endothelial quiescence by FOXO-regulated metabolites. Nat Cell Biol 23:413–423. 10.1038/s41556-021-00637-6 PMC8032556 33795871

[23] AlZaim I, Hassan MN, Schröter M et al (2026) Defining the vascular niche of human adipose tissue across metabolic states. Nat Metab 8:722–740. 10.1038/s42255-026-01475-2 41820564

[24] Nwadozi E, Roudier E, Rullman E et al (2016) Endothelial FoxO proteins impair insulin sensitivity and restrain muscle angiogenesis in response to a high-fat diet. FASEB J 30:3039–3052. 10.1096/fj.201600245R 27235148

[25] Lindhorst A, Raulien N, Wieghofer P et al (2021) Adipocyte death triggers a pro-inflammatory response and induces metabolic activation of resident macrophages. Cell Death Dis 12:579. 10.1038/s41419-021-03872-9 PMC8179930 34091595

[26] Bonner JS, Lantier L, Hasenour CM et al (2013) Muscle-specific vascular endothelial growth factor deletion induces muscle capillary rarefaction creating muscle insulin resistance. Diabetes 62:572–580. 10.2337/db12-0354 PMC3554359 23002035

[27] Prior SJ, McKenzie MJ, Joseph LJ et al (2009) Reduced skeletal muscle capillarization and glucose intolerance. Microcirculation 16:203–212. 10.1080/10739680802502423 PMC2990692 19225985

[28] Paik J-H, Kollipara R, Chu G et al (2007) FoxOs are lineage-restricted redundant tumor suppressors and regulate endothelial cell homeostasis. Cell 128:309–323. 10.1016/j.cell.2006.12.029 PMC1855089 17254969

[29] Ueda S, Tominaga T, Ochi A et al (2021) TGF-β1 is involved in senescence-related pathways in glomerular endothelial cells via p16 translocation and p21 induction. Sci Rep 11:21643. 10.1038/s41598-021-01150-4 PMC8569175 34737348

[30] Sun D, Chen S, Li S et al (2022) Enhancement of glycolysis-dependent DNA repair regulated by FOXO1 knockdown via PFKFB3 attenuates hyperglycemia-induced endothelial oxidative stress injury. Redox Biol 59:102589. 10.1016/j.redox.2022.102589 PMC9803794 36577299

[31] Kluge MA, Fetterman JL, Vita JA (2013) Mitochondria and endothelial function. Circ Res 112:1171–1188. 10.1161/CIRCRESAHA.111.300233 PMC3700369 23580773

[32] Cheng Z, Guo S, Copps K et al (2009) Foxo1 integrates insulin signaling with mitochondrial function in the liver. Nat Med 15:1307–1311. 10.1038/nm.2049 PMC3994712 19838201

[33] Li B, Ming H, Qin S et al (2025) Redox regulation: mechanisms, biology and therapeutic targets in diseases. Sig Transduct Target Ther 10:72. 10.1038/s41392-024-02095-6 PMC11885647 40050273

[34] Collins PL, Purman C, Porter SI et al (2020) DNA double-strand breaks induce H2Ax phosphorylation domains in a contact-dependent manner. Nat Commun 11:3158. 10.1038/s41467-020-16926-x PMC7308414 32572033

[35] Sullivan KD, Galbraith MD, Andrysik Z, Espinosa JM (2018) Mechanisms of transcriptional regulation by p53. Cell Death Differ 25:133–143. 10.1038/cdd.2017.174 PMC5729533 29125602

[36] Hollander MC, Alamo I, Jackman J et al (1993) Analysis of the mammalian *gadd45* gene and its response to DNA damage. J Biol Chem 268:24385–24393 8226988

[37] Wu Y, Pegoraro AF, Weitz DA et al (2022) The correlation between cell and nucleus size is explained by an eukaryotic cell growth model. PLoS Comput Biol 18:e1009400. 10.1371/journal.pcbi.1009400 PMC8893647 35180215

[38] Yang X, Wang L, Liu Y et al (2026) Endothelial cell senescence: a core mechanism in the development of cardiovascular metabolic diseases. Int J Cardiol. 10.1016/j.ijcard.2026.134276 41795854

[39] Kim SY, Ko YS, Park J et al (2016) Forkhead transcription factor FOXO1 inhibits angiogenesis in gastric cancer in relation to SIRT1. Cancer Res Treat 48:345–354. 10.4143/crt.2014.247 PMC4720104 25761483

[40] Niimi K, Kohara M, Sedoh E et al (2020) FOXO1 regulates developmental lymphangiogenesis by upregulating CXCR4 in the mouse-tail dermis. Development 147:dev181545. 10.1242/dev.181545 31852686

[41] Shi Y, Fan S, Wang D et al (2018) FOXO1 inhibition potentiates endothelial angiogenic functions in diabetes via suppression of ROCK1/Drp1-mediated mitochondrial fission. Biochimica et Biophysica Acta (BBA)—Molecular Basis Disease 1864:2481–2494. 10.1016/j.bbadis.2018.04.005 29654945

[42] Al-Radi O, Ingelshed K, Eichhorn L et al (2025) Pharmacological activation of p53 induces dose-dependent changes in endothelial cell fate during angiogenic sprouting. Cell Death Dis 16:883. 10.1038/s41419-025-08292-7 PMC12698774 41360924

[43] Damacena de Angelis C, Endoni BT, Nuno D et al (2022) Sex‐specific differences in endothelial function are driven by divergent mitochondrial Ca2+ handling. J Am Heart Assoc 11:e023912. 10.1161/JAHA.121.023912 PMC9333382 35766269

[44] Hägg S, Jylhävä J, Wang Y et al (2021) Deciphering the genetic and epidemiological landscape of mitochondrial DNA abundance. Hum Genet 140:849–861. 10.1007/s00439-020-02249-w PMC8099832 33385171

[45] Demarest TG, McCarthy MM (2015) Sex differences in mitochondrial (dys)function: implications for neuroprotection. J Bioenerg Biomembr 47:173–188. 10.1007/s10863-014-9583-7 PMC4988325 25293493

[46] Chen H, Chomyn A, Chan DC (2005) Disruption of fusion results in mitochondrial heterogeneity and dysfunction. J Biol Chem 280:26185–26192. 10.1074/jbc.M503062200 15899901

[47] Machiela E, Liontis T, Dues DJ et al (2020) Disruption of mitochondrial dynamics increases stress resistance through activation of multiple stress response pathways. FASEB J 34:8475–8492. 10.1096/fj.201903235R PMC7313680 32385951

[48] Bhatti JS, Bhatti GK, Reddy PH (2017) Mitochondrial dysfunction and oxidative stress in metabolic disorders—a step towards mitochondria based therapeutic strategies. Biochimica et Biophysica Acta (BBA) - Molecular Basis Disease 1863:1066–1077. 10.1016/j.bbadis.2016.11.010 PMC5423868 27836629

[49] Martini H, Passos JF (2023) Cellular senescence: all roads lead to mitochondria. FEBS J 290:1186–1202. 10.1111/febs.16361 PMC9296701 35048548

[50] Rodier F, Coppé J-P, Patil CK et al (2009) Persistent DNA damage signalling triggers senescence-associated inflammatory cytokine secretion. Nat Cell Biol 11:973–979. 10.1038/ncb1909 PMC2743561 19597488

[51] Yousefzadeh MJ, Zhao J, Bukata C et al (2020) Tissue specificity of senescent cell accumulation during physiologic and accelerated aging of mice. Aging Cell 19:e13094. 10.1111/acel.13094 PMC7059165 31981461

[52] Quarder A, Talbi K, Bartmann H et al (2026) Sex and stimulus-specific differences in endothelial cell senescence under hormone-free conditions. Am J Physiol Heart Circ Physiol 330:H515–H523. 10.1152/ajpheart.00646.2025 41512281

[53] Tarvainen S, Wirth G, Juusola G et al (2024) Critical limb-threatening ischaemia and microvascular transformation: clinical implications. Eur Heart J 45:255–264. 10.1093/eurheartj/ehad562 PMC10821383 37634134

[54] Barinda AJ, Ikeda K, Nugroho DB et al (2020) Endothelial progeria induces adipose tissue senescence and impairs insulin sensitivity through senescence associated secretory phenotype. Nat Commun 11:481. 10.1038/s41467-020-14387-w PMC6981212 31980643

[55] Rudnicki M, Pislaru A, Haas TL (2022) Quantitative methods to assess adipose vasculature. In: Benest AV (ed) Angiogenesis: methods and protocols. Springer US, New York, NY, pp 201–221 10.1007/978-1-0716-2059-5_16 35099739

[56] Subramanian A, Tamayo P, Mootha VK et al (2005) Gene set enrichment analysis: a knowledge-based approach for interpreting genome-wide expression profiles. Proc Natl Acad Sci U S A 102:15545–15550. 10.1073/pnas.0506580102 PMC1239896 16199517

[57] Hao Y, Stuart T, Kowalski MH et al (2024) Dictionary learning for integrative, multimodal and scalable single-cell analysis. Nat Biotechnol 42:293–304. 10.1038/s41587-023-01767-y PMC10928517 37231261

[58] Chaudhry A, Shi R, Luciani DS (2020) A pipeline for multidimensional confocal analysis of mitochondrial morphology, function, and dynamics in pancreatic β-cells. Am J Physiol Endocrinol Metab 318:E87–E101. 10.1152/ajpendo.00457.2019 PMC7052579 31846372

