# Supplementary material for: Female-specific reliance on endothelial FoxO1 governs vascular resilience and metabolic adaptation to obesity
Source: Angiogenesis. 2026 Oct 22;29(4):79. doi: 10.1007/s10456-026-10093-1 (PMC13690923; doi:10.1007/s10456-026-10093-1)
Supplement: Supplementary file 1 [file 10456_2026_10093_MOESM2_ESM.pdf]

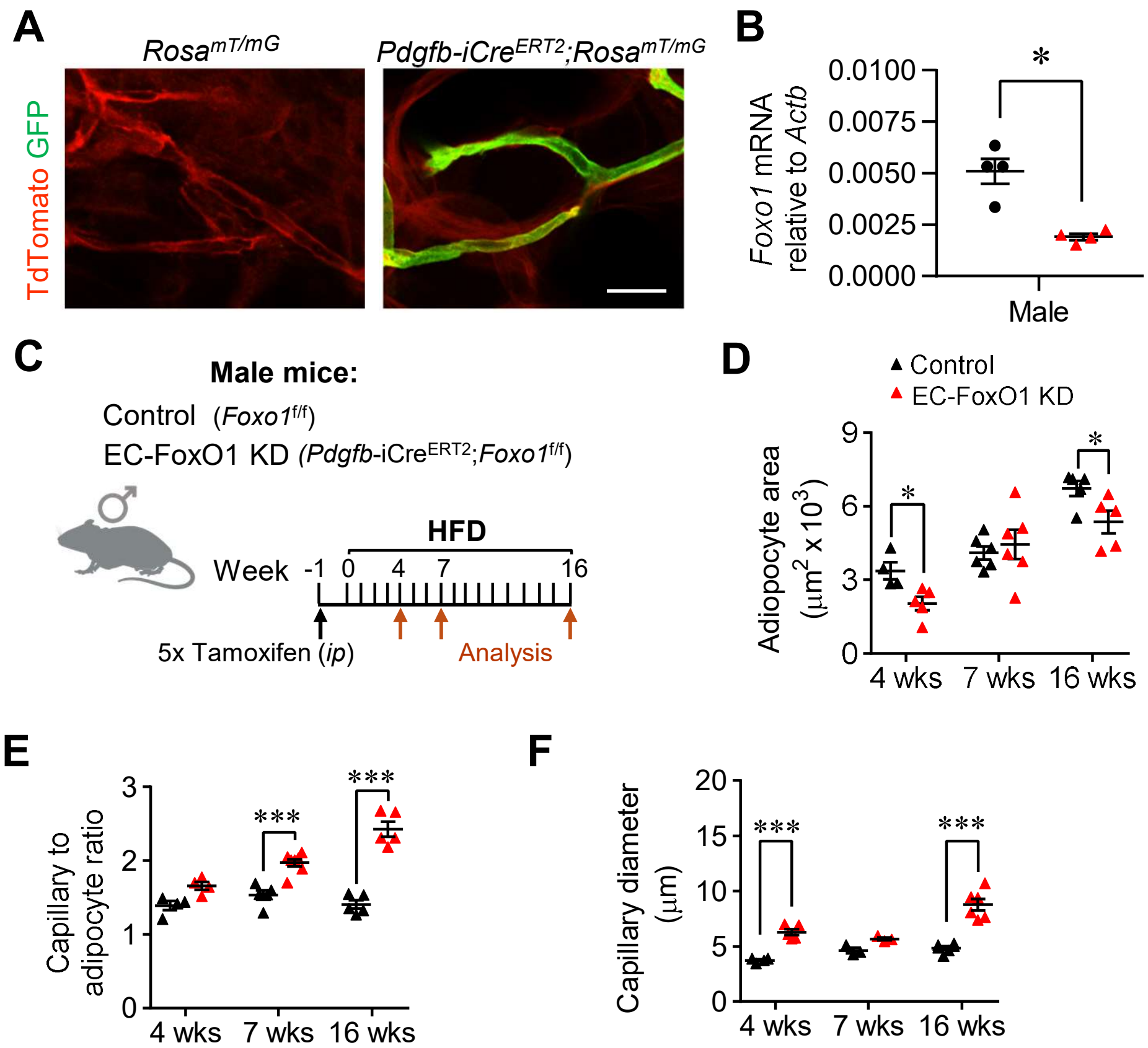

Fig S1. Capillary content increases with EC-FoxO1 knockdown in HFD-fed male mice. A pWAT images of tamoxifen-injected *Rosa*$^{mT/mG}$ and *Pdgfb-iCre*^{ERT2}*;Rosa*$^{mT/mG}$ male mice illustrate the endothelial-specificity of Cre recombinase activity. Green: GFP; Red: tdTomato. Scale bar=20 µm. **B** *Foxo1* mRNA relative to *Actb* ($2^{-\Delta CT}$) in male EC isolated from white adipose of 7-week HF fed Control and EC-FoxO1 KD mice (n=4). **C** Control and EC-FoxO1 KD male mice were fed High Fat Diet (HFD) for 4-, 7- or 16- weeks following tamoxifen injection. Quantification of **D** adipocyte areas, **E** capillary to adipocyte ratios and **F** adipose capillary diameters in within pWAT from these HFD-fed male mice (16-wk data from [9]. Data points reflect individual mice and are plotted with the group mean ± SEM. Data for each time point were analyzed by Students t-test. *P<0.05; **P<0.01; ***P<0.001. Schematic created in BioRender. Rudnicki, M. (2026) https://BioRender.com/y2m3teo

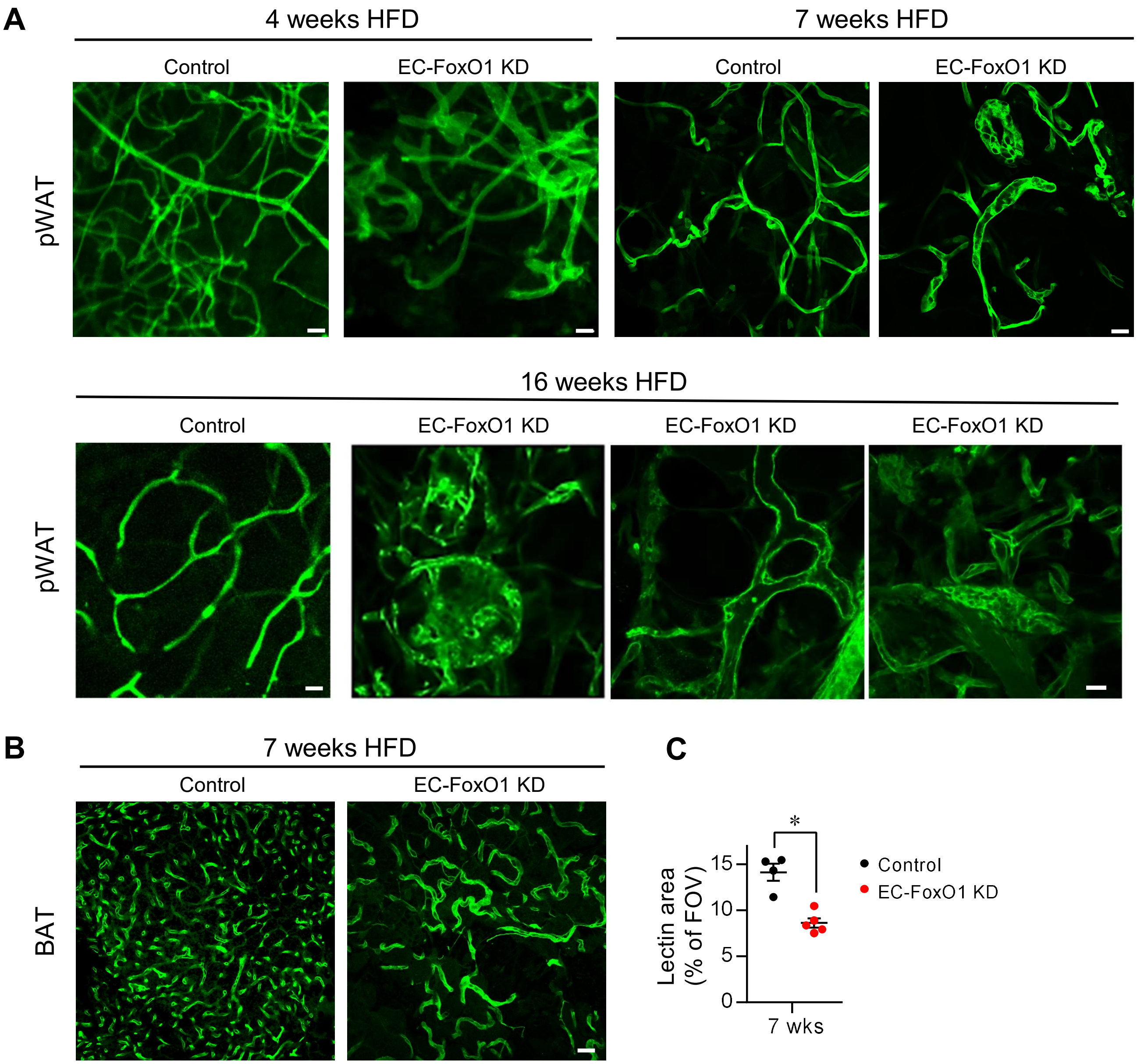

Fig S2. Capillary dysmorphia in EC-FoxO1 KD female mice. Control and EC FoxO1 KD female mice were fed High Fat Diet (HFD) for 4, 7, or 16 weeks following tamoxifen injection. **A** Confocal whole mount imaging of perigonadal white adipose tissue (pWAT) stained with *G. simplificolia* (green) to detect capillaries. Scale bar = 20 µm). **B** Confocal whole mount imaging of brown adipose tissue (BAT) stained with *G. simplificolia* (green) to detect capillaries. Scale bar = 20 µm). **C** Quantification of capillary density in BAT of female mice after 7 weeks HFD. Data points reflect individual mice and are plotted with the group mean ± SEM. Data were analyzed by unpaired Students t-test. $^{*}P<0.05$

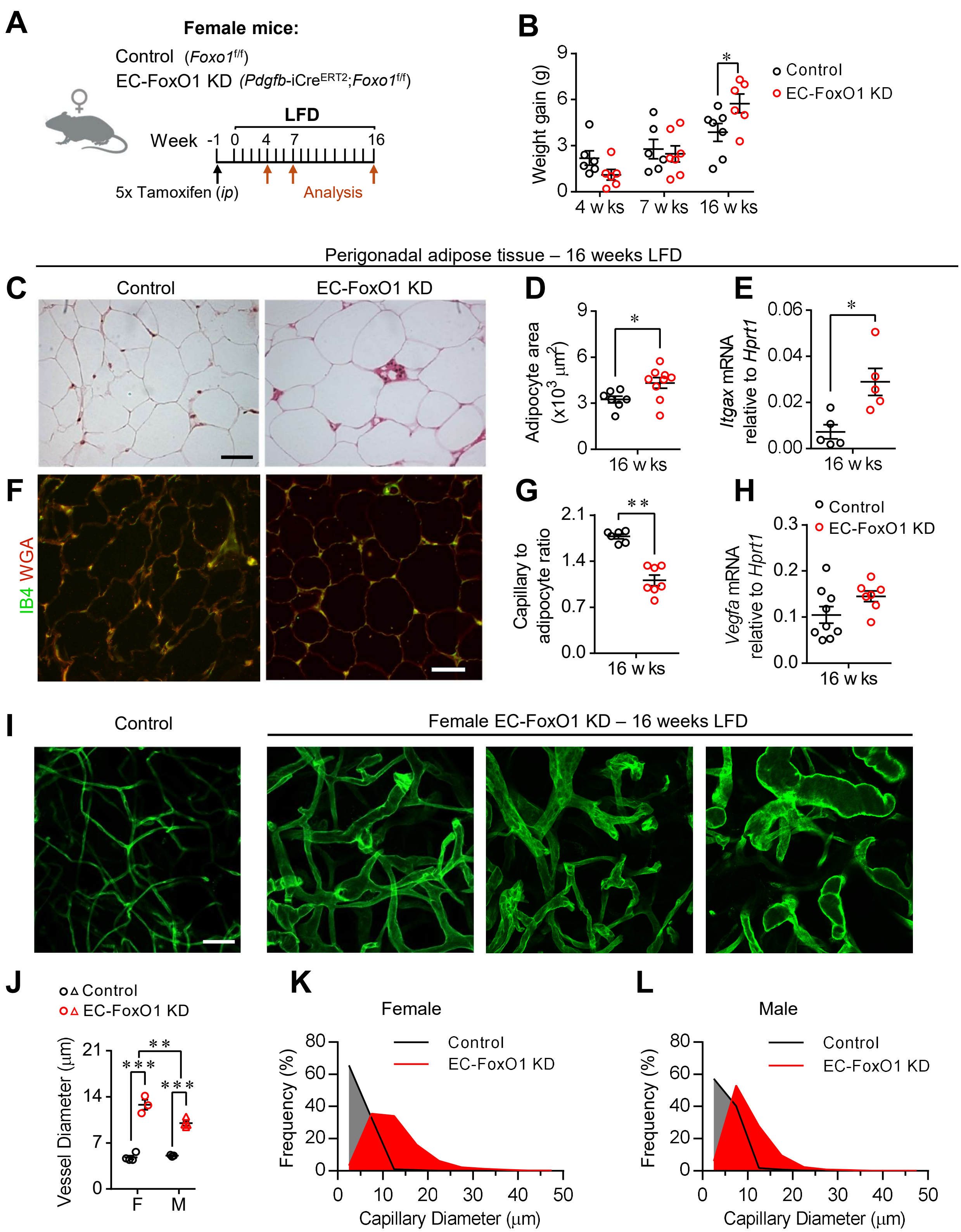

Figure S3. Adipose capillary dysmorphia and diameter enlargement in female mice following EC-FoxO1 knockdown are diet-independent. A Control and EC FoxO1 KD female mice were fed low fat diet (LFD) for 16 weeks following tamoxifen injection. **B** Weight gain over the 16 weeks of LFD. **C** Hematoxylin and eosin-stained images of perigonadal adipose tissue from 16-week LFD-fed female mice. Scale bar=50 μm **D** Mean adipocyte cross-sectional areas. **E** *Itgax* mRNA relative to *Hprt1* ($2^{-\Delta CT}$) in perigonadal adipose tissue of 16-week LFD-fed female mice (n=5). **F** *G. simplicifolia* lectin-FITC (green) and Rhodamine Wheat germ agglutinin (red) staining of adipose tissue cross-sections to label capillaries and basement membranes, respectively. Scale bar=100 μm. **G** Quantification of capillary to adipocyte ratios. **H** *Vegfa* mRNA relative to *Hprt1* ($2^{-\Delta CT}$) in perigonadal adipose tissue of 16-week LFD-fed female mice (n=5). **I** Whole mount confocal imaging of female adipose vasculature (stained with *G. simplicifolia* lectin-FITC). Scale bar = 50 μm **J** Quantification of capillary diameters of 16-week LFD-fed female and male Control and EC-FoxO1 KD mice. **K-L** Frequency histogram summarizing capillary diameter distributions. Except for the frequency histograms, data points reflect individual mice and are plotted with the group mean ± SEM (except in **K-L**). Data for each time point were analyzed by Students t-test. *P<0.05; **P<0.01; ***P<0.001. Schematic created in BioRender. Rudnicki, M. (2026) https://BioRender.com/gtbkoy3

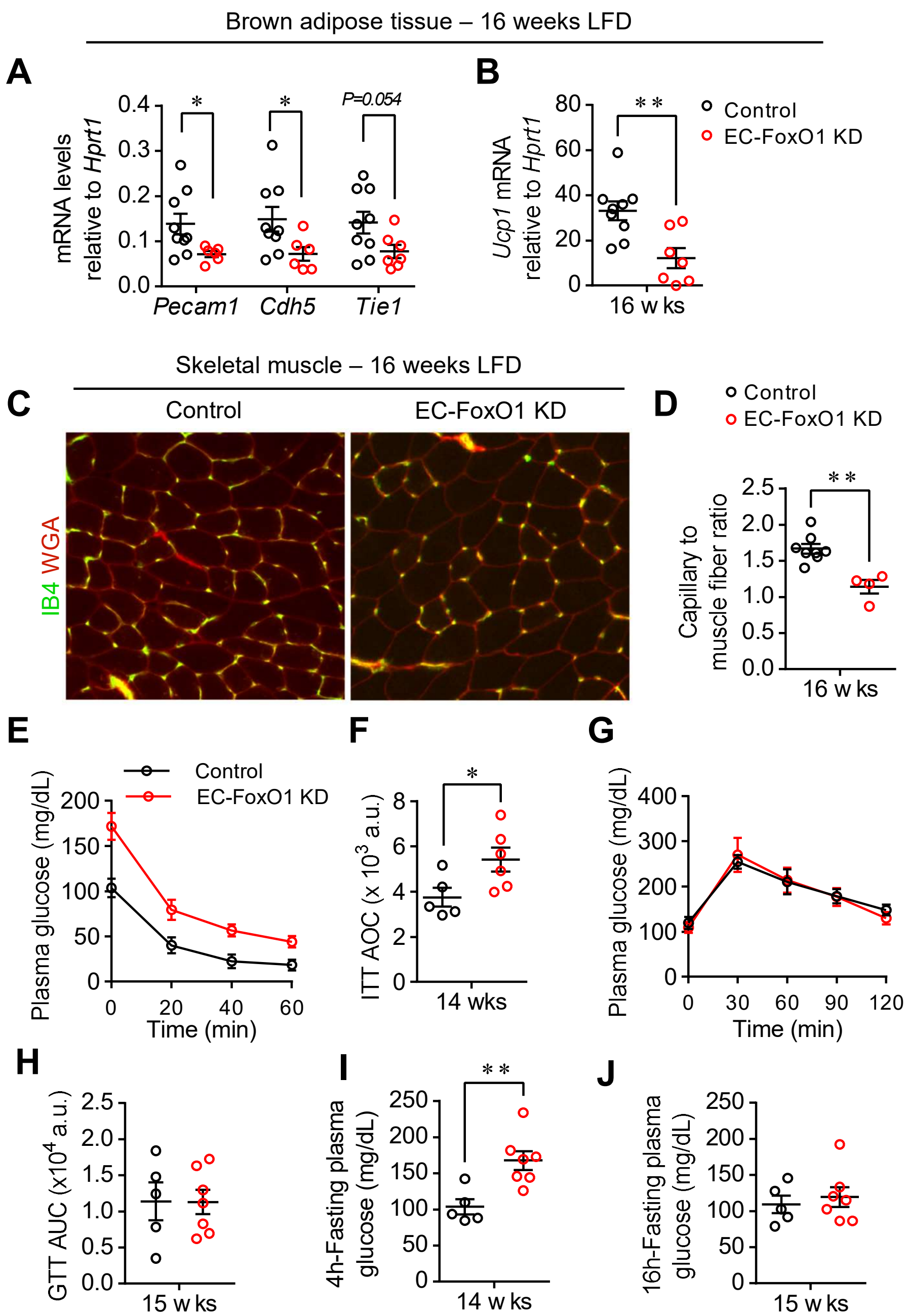

Fig S4. Capillary content decreases in brown adipose and skeletal muscle with EC-FoxO1 knockdown in LFD-fed female mice. Control and EC-FoxO1 KD female mice were fed low fat diet (LFD) for 16 wks. **A-B** Endothelial marker genes and *Ucp1* mRNA relative to *Hprt1* ($2^{-\Delta CT}$) from female brown adipose tissue. **C** Cross-sections of female EDL muscle stained with *G. simplicifolia* lectin-FITC (IB4; green) and Rhodamine Wheat Germ Agglutinin (WGA; red) to visualize capillaries and muscle fibers, respectively. Scale bar=100 µm **D** Quantification of capillary to fiber ratios. **E** Insulin sensitivity was assessed by intraperitoneal insulin tolerance test (ITT). **F** ITT area over the curve (AOC). **G** Glucose tolerance was assessed by intraperitoneal glucose tolerance test (GTT). **H** GTT area under the curve (AUC). **I-J** Plasma glucose levels after 4 or 16 hours fasting. Data points reflect individual mice and are plotted with the group mean ± SEM. Data were analyzed by Students *t*-tests. *$P<0.05$; **$P<0.01$.

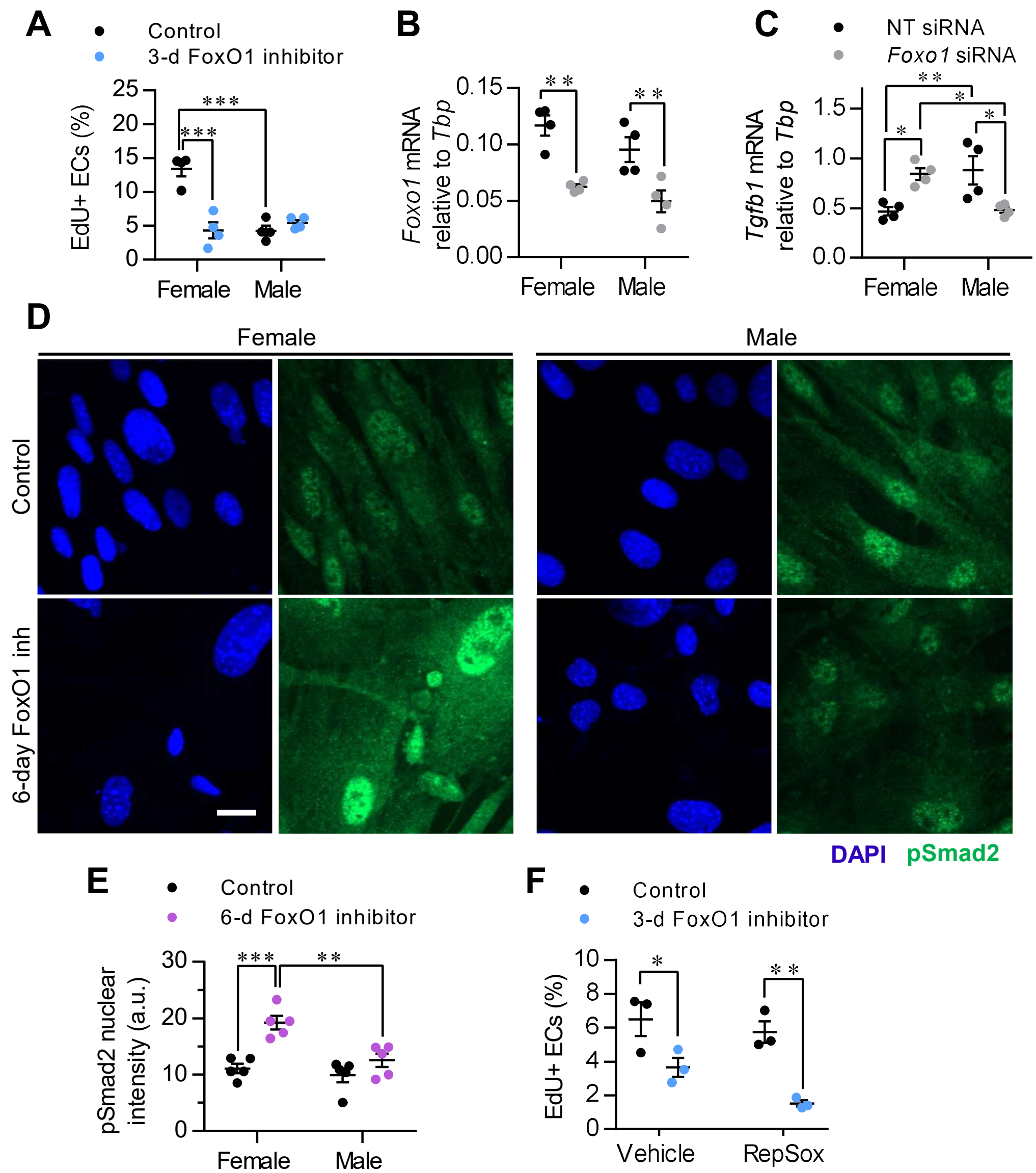

Fig S5. TGFβ signaling is amplified by FoxO1 inhibition in female EC but does not mediate the lowered proliferation. A Cultured female and male EC were treated with vehicle or FoxO1 inhibitor (AS1842856, 1 μM) for 3-days. EdU incorporation during the final 4 hours was quantified as a percent of total cell number. **B-C** *Foxo1* and *Tgfb1* mRNA in cultured adipose EC treated with non-targeting (NT) siRNA or *Foxo1* siRNA for 24 hours, normalized to housekeeping *Tbp* ($2^{-\Delta CT}$). **D** Phospho-(p)Smad2 (green) immunostaining and DAPI (blue). Scale bar=20 μm**. E** Quantification of nuclear pSmad2 relative fluorescence intensity (a.u., arbitrary units) (n=5). **F** EdU incorporation in female EC that were treated for 3 days with vehicle or TGFBR2 inhibitor RepSox (5 μM), alone or with FoxO1 inhibitor (1 μM). Individual data points reflect independent experiments. Data were analyzed by 2-way ANOVA (Treatment x Sex) with Sidak post-hoc tests when main effects were significant. **P*<0.05; ***P*<0.01; ****P*<0.001.

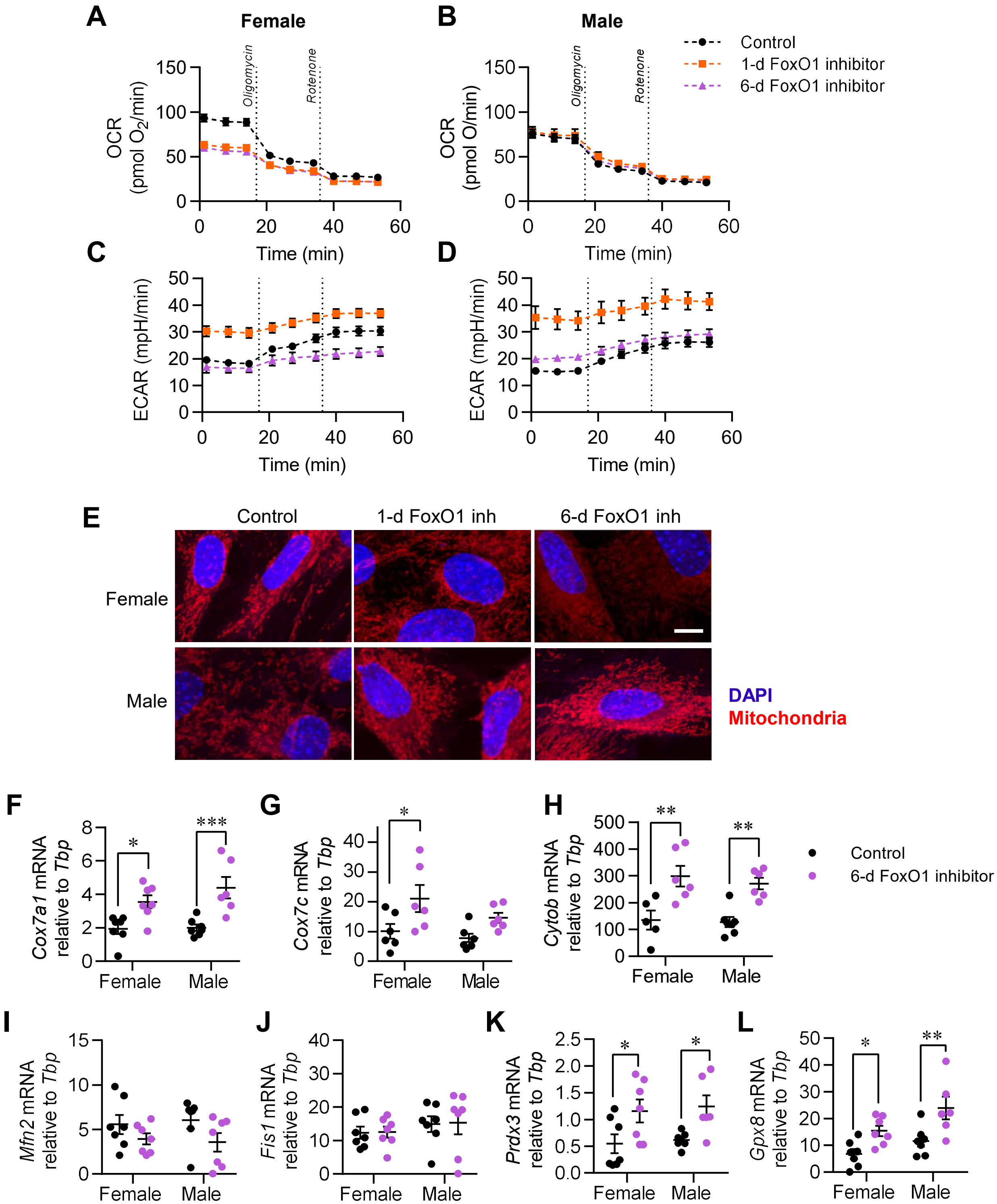

Fig S6. Effects of FoxO1 inhibition on metabolic function, and the expression of mitochondrial and antioxidant genes. Female and male mouse adipose EC were treated for 1 or 6 days (1- or 6-d) with FoxO1 inhibitor (AS1842856, 1 µM) or vehicle. Cellular metabolism in female and male EC was assessed by Seahorse (ATP production assay), with oxygen consumption rates (OCR) shown in **A,B** and extracellular acidification rates (ECAR) in **C,D**. Data were normalized to account for well-to-well variances in cell density. **E** EC stained with MitoBrilliant (red) and DAPI (blue) to visualize mitochondrial networks. Scale bar=10 µm**.** mRNA level of **F-H** mitochondrial genes *Cox7a1*, *Cox7c*, and *Cytob,* **I,J** mitochondrial dynamics genes *Mfn2* and *Fis1* and **K,L** antioxidant genes *Prdx3* and *Gpx8* were normalized to housekeeping *Tbp* ($2^{-\Delta CT}$) (Female n=5-7, Male n=6). Individual data points reflect independent experiments. Data in E-I were analyzed by 2-way ANOVA (Treatment x Sex) with Sidak post-hoc tests when main effects were significant. **P*<0.05; ***P*<0.01; ****P*<0.001.

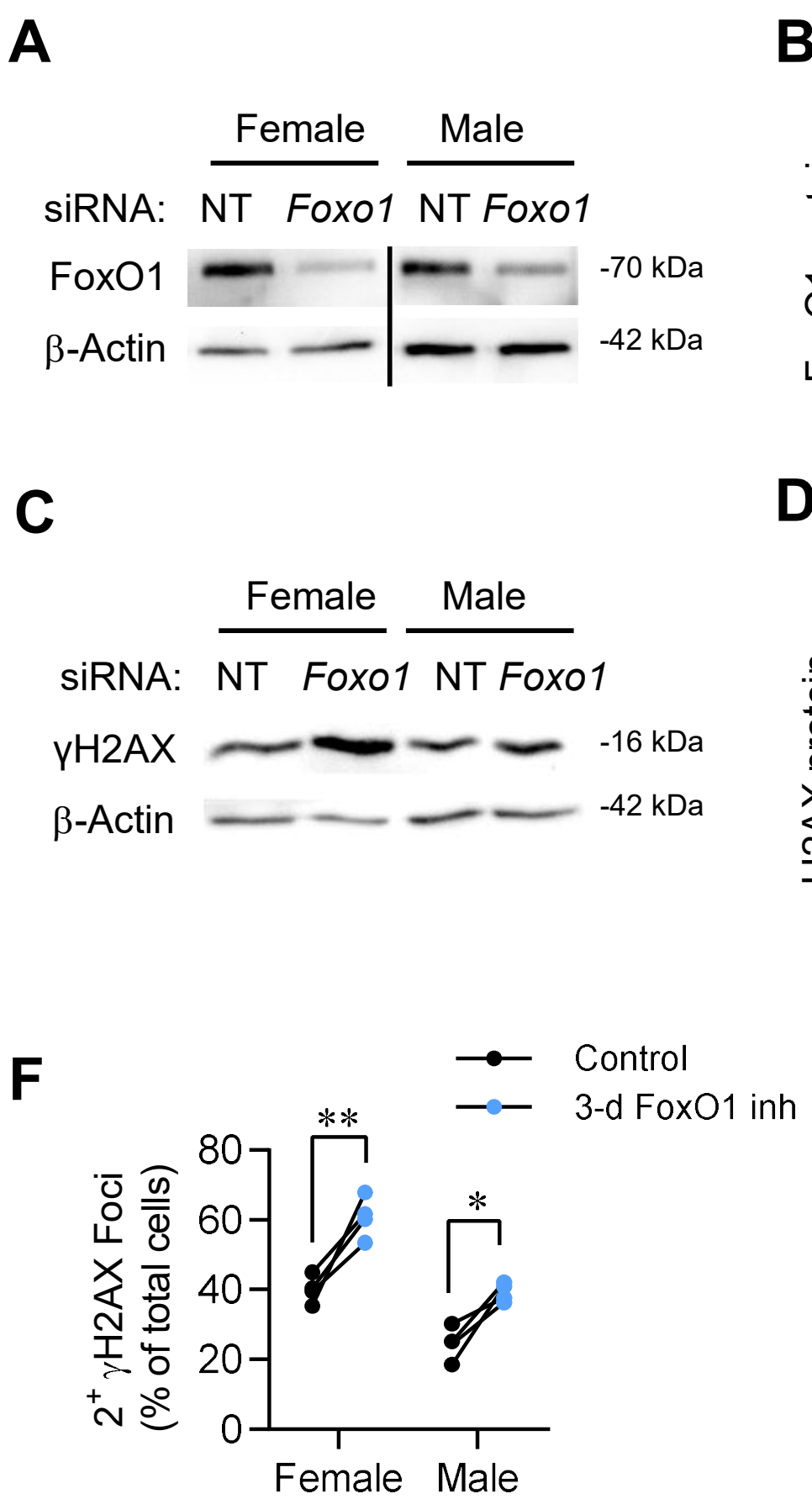

Fig S7. DNA damage increases after FoxO1 silencing or inhibition in female EC. EC were treated with non-targeting (NT) siRNA or *Foxo1* siRNA for 3-days. **A** Western blot of FoxO1 and **B** Quantification of FoxO1 protein relative to ***β***-actin (n=5). **C** Western blot of γH2AX levels and **D** Quantification of γH2AX relative to ***β***-actin (n=5). **E** Nucleus area in female and male EC following siRNA treatment **F** Quantification of γH2AX loci and **G** Nucleus size in EC following 3-days of FoxO1 inhibition. Vertical line in **A** indicates that the image of the original blots was cropped to remove lanes that contained other treatments. Data were analyzed by 2-way ANOVA (Treatment x Sex) with Sidak post-hoc tests when main effects were significant. *P<0.05; **P<0.01.
