# Supplementary material for: Female-specific reliance on endothelial FoxO1 governs vascular resilience and metabolic adaptation to obesity
Source: Angiogenesis. 2026 Oct 22;29(4):79. doi: 10.1007/s10456-026-10093-1 (PMC13690923; doi:10.1007/s10456-026-10093-1)
Supplement: Supplementary file 2 [file 10456_2026_10093_MOESM3_ESM.pdf]

Table S1. Tissue weights of female mice after 16 weeks of LFD or HFD diet

	LFD		HFD	
	**Control**	**EC-FoxO1 KD**	**Control**	**EC-FoxO1 KD**
Final body weight (g)	26.4 ± 0.7	30.0 ± 2.0	31.2 ± 1.4	39.4 ± 5.2#
Body weight gain (wk 14-0) (g)	4.2 ± 0.6	5.6 ± 0.8	6.4 ± 1.0	15.6 ± 4.4*
pWAT (g)	0.9 ± 0.1	1.6 ± 0.2*	1.2 ± 0.2	2.8 ± 0.6**
rWAT (g) $	0.4 ± 0.06	0.9 ± 0.2	0.6 ± 0.1	1.2 ± 0.3
Subcutaneous WAT (g)	0.9 ± 0.3	1.7 ± 0.3	1.4 ± 0.2	4.3 ± 1.6
BAT (mg)	128.8 ± 5.8	202.3 ± 13.2*	109.3 ± 12.4	203.5 ± 21.6***
Liver (g)	1.0 ± 0.1	1.2 ± 0.1	1.0 ± 0.1	1.4 ± 0.2*
Heart (mg)	100.2 ± 3.6	107.7 ± 6.6	114.2 ± 6.8	117.6 ± 7.9
Gastrocnemius (mg)	96.2 ± 3.2	96.9 ± 4.6	102.0 ± 4.6	100.9 ± 5.6
Soleus (mg)	5.8 ± 0.3	8.2 ± 0.6	6.7 ± 0.3	7.3 ± 0.5
Tibialis anterior (mg)	37.0 ± 1.1	32.3 ± 3.2	37.6 ± 1.9	37.4 ± 1.7
Exensor digitorum longus (mg)	9.4 ± 0.8	8.6 ± 0.7	8.9 ± 0.4	9.1 ± 0.4
Plantaris (mg)	11.1 ± 1.1	12.9 ± 0.7	11.3 ± 0.5	13.2 ± 0.4

LFD-low fat diet; HFD-high fat diet; pWAT- perigonadal white adipose tissue; rWAT - retroperitoneal white adipose tissue; BAT -brown adipose tissue

Data are expressed as mean ± S.E.M., n=5-9 per group (except Subcutaneous WAT Control groups, n=3) $ Main effect of genotype; * $P < 0.05$, ** $P<0.01$, *** $P<0.001$ EC-FoxO1 KD *vs* Control group; # $P<0.05$ HF *vs* NC within same genotype; 2-way ANOVA with Sidak's post-hoc

Table S3: List of Antibodies and suppliers

Antibody	Dilution	Supplier	Catalogue #
Cyclin-dependent kinase inhibitor 1a (p21)	1:100 (IF)	Santa Cruz Biotech	sc-6246
Cyclin-dependent kinase inhibitor 2a (p16 INK4A)	1:100 (IF) 1:1000 (W)	Cell Signaling	82172
4-Hydroxynonenal (4-HNE)	1:100 (IF)	R&D systems	MAB3249
Phosphorylated H2A histone family member X (γH2AX)	1:400 (IF); 1:1000 (W)	Cell Signaling	2577
Phospho-Smad2	1:100 (IF)	Cell Signaling	3101
p53	1:100 (IF)	Santa Cruz	sc-6243
FoxO1	1:1000 (W)	Cell Signaling	2880
Ki67	1:100 (IF)	Abclonal	A21861
Ribosomal protein S6	1:1000 (W)	Proteintech	80208-1-RR
Phosphorylated ribosomal protein S6	1:1000 (W)	Proteintech	29223-1-AP
Beta-actin	1:5000 (W)	Santa Cruz	sc-47778
Cy™3 AffiniPure® Goat Anti-Rabbit IgG (H+L)	1:200 (IF)	Jackson ImmunoResearch	111-165-003
Cy™3 AffiniPure® Goat Anti-Mouse IgG (H+L)	1:200 (IF)	Jackson ImmunoResearch	115-165-003
Fluorescein (FITC) AffiniPure® $F(ab')_2$ Fragment Goat Anti-Mouse IgG (H+L)	1:200 (IF)	Jackson ImmunoResearch	115-096-003
Fluorescein (FITC) AffiniPure® $F(ab')_2$ Fragment Goat Anti-Rabbit IgG (H+L)	1:200 (IF)	Jackson ImmunoResearch	111-096-003
Goat Anti-Rabbit IgG	1:10,000 (W)	Jackson ImmunoResearch	111-035-003
Goat Anti-Mouse IgG	1:10,000 (W)	Jackson ImmunoResearch	115-035-003
G. simplicifolia-FITC lectin	1:100-200 (IF)	Vector Labs	FL-1101
Wheat germ agglutinin-Rhodamine	1:200-400 (IF)	Vector Labs	RL-1022

IF-immunofluorescence; W-Western blot

Table S4: List of Taqman Primer/Probe sets

Target Gene	Assay Code
Actb	Mm04394036_g1
Cdh5	Mm00486938_m1
Cox7a1	Mm00438297_g1
Cox7c	Mm01545088_g1
Fis1	Mm00481580_m1
Foxo1	Mm00490671_m1
Gadd45a	Mm00432802_m1
Gpx8	Mm00510156_m1
Hprt1	Mm00446968_m1
Itgax	Mm00498701_m1
Mfn2	Mm00500120_m1
Mki67	Mm01278617_m1
Mt-cytb	Mm04225271_g1
Pecam1	Mm00476712_m1
Prdx3	Mm00545844_m1
Tbp	Mm00446973_m1
Tgfb1	Mm01178820_m1
Tie1	Mm00441786_m1
Ucp1	Mm01244861_m1
Vegfa	Mm00437306_m1
